# A Focused Review of Synthetic Applications of Lawesson’s Reagent in Organic Synthesis

**DOI:** 10.3390/molecules26226937

**Published:** 2021-11-17

**Authors:** Hena Khatoon, Emilia Abdulmalek

**Affiliations:** 1 Department of Chemistry, Faculty of Science, Universiti Putra Malaysia, Serdang 43400, Selangor, Malaysia; 2 Integrated Chemical BioPhysics Research, Faculty of Science, Universiti Putra Malaysia, Serdang 43400, Selangor, Malaysia

**Keywords:** Lawesson’s reagent, organic synthesis, thio analogs, biological applications

## Abstract

Lawesson’s reagent (LR) is a well-known classic example of a compound with unique construction and unusual chemical behavior, with a wide range of applications in synthetic organic chemistry. Its main functions were rounded for the thionation of various carbonyl groups in the early days, with exemplary results. However, the role of Lawesson’s reagent in synthesis has changed drastically, and now its use can help the chemistry community to understand innovative ideas. These include constructing biologically valuable heterocycles, coupling reactions, and the thionation of natural compounds. The ease of availability and the convenient usage of LR as a thionating agent made us compile a review on the new diverse applications on some common functional groups, such as ketones, esters, amides, alcohols, and carboxylic acids, with biological applications. Since the applications of LR are now diverse, we have also included some new classes of heterocycles such as thiazepines, phosphine sulfides, thiophenes, and organothiophosphorus compounds. Thionation of some biologically essential steroids and terpenoids has also been compiled. This review discusses the recent insights into and synthetic applications of this famous reagent from 2009 to January 2021.

## 1. Introduction

**Figure 1:**
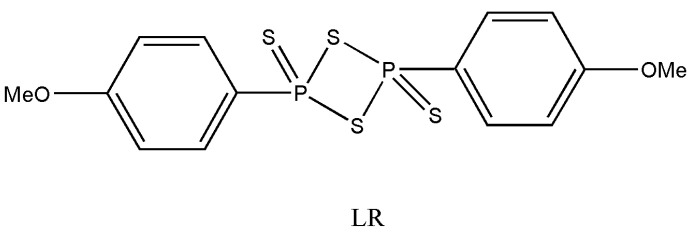
2,4-bis(p-methoxyphenyl)-1,3-dithiadiphosphetane-2,4-disulfide or Lawesson’s reagent (LR).

The carbonyl group’s conversion to thiocarbonyl has always been in the interest of synthetic organic chemists for many years. Two essential reagents, namely phosphorus pentasulfide (P_4_S_10_) and Lawesson’s reagent (LR) (Figure 1), are the most widely used agents for such a transformation. However, as the most commonly used reagent since the beginning of the 20th century, LR has gained the upper hand with essential applications in synthetic organic chemistry [1,2,3,4,5,6].

The main advantage of LR over P_4_S_10_ is its lesser requirement and shorter reaction time, primarily if reactions are carried out using microwave irradiation techniques. In addition, the high yields, convenient handling, availability, and soft thionation reactions are also unique LR benefits.

Lawesson’s reagents were first systematically studied by Lawesson and co-workers in 1978, mainly for carbonyl groups’ transition to thiocarbonyls [7,8,9]. LR is commercially available and widely used in organic synthesis. However, it has been reviewed that LR becomes unstable above 110 °C and decomposes or polymerizes slowly [10].

The reactivity of hydroxyl and carboxyl groups towards LR was reported by Nishio et al. [11]. The authors described that the hydroxyl groups are the most reactive, whereas the esters are the least. The reactivity series were presented as follows:

Hydroxyl > Amide > Ketone > Esters(1)

The unique properties and synthesis of thio analogs using LR have been highlighted by Ozturk et al. in their comprehensive review in 2007 [10]. In 2017, Saeed et al. reviewed alkaloids’ thionation, focusing primarily on carbonyl groups [12].

The thionation of complex structures using LR, for example, cyclic peptides such as [Tyr-3-Ψ-(CS-NH)-Ala-4; Tyr-6-Ψ-(CSNH)-d-Ala-1]RA-VII [13], steroids such as 17b-hydroxy-3-thioxo-4-aza-5-androstane [14], or nucleosides such as 1-(2,3,5-tri-O-benzoyl-b-d-ribofuranosyl)-4-thioxothieno[3,2-d]pyrimidin-2-one [15], is another well-known application.

LR is also widely used to form organothiophosphorus compounds, a subclass of organophosphorus compounds with many applications in the pesticide industry, medical fields, and oil additives. Furthermore, these compounds have been identified as anticancer, antiviral, cardioprotective therapeutics, and acetylcholine esterase inhibitors in medicinal chemistry [16].

Lawesson’s reagent was also systematically reviewed by Kayukova et al. [17], emphasizing primary thionation of carbonyl carbon, rearrangement reactions with or without sulfur insertion, and formation of organophosphorus compounds with specific biological applications.

**Scheme 1:**
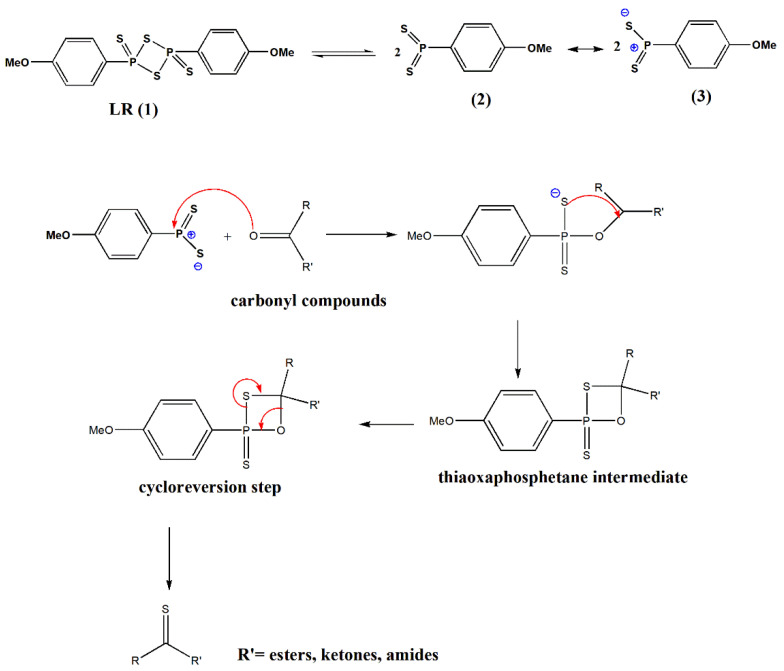
Mechanism of the thionation reaction using Lawesson’s reagent (1).

The mechanism of thionation reactions using LR has been portrayed in the literature [6,17]. Lawesson’s reagent in solution exists in equilibrium with more reactive dithiophosphine ylide (2, 3). These reactive species (2, 3) can easily interact with carbonyl to afford the thiaoxaphosphetane intermediate (Scheme 1). The driving force creates stable P=O bonds in a cycloreversion step similar to the mechanisms known for the Wittig reaction [6].

The reaction rate is faster for ketones, amides, lactones, and lactams than esters, which is comparatively unreactive and necessitates special conditions for suitable transformations.

Considering the insight into the nature of LR and its diverse synthetic applications, we wrote this synthetic review to the best of our knowledge. Here, we investigated the developments in applying this powerful reagent and provided an overview of synthetic reactions using Lawesson’s reagent from 2009 to January 2021.

## 2. Synthesis of Some Biologically Essential Thioketones

Ketones can be easily converted by O/S exchange using Lawesson’s reagent (LR), which forms comparatively stable aromatic thioketones; therefore, it has been studied extensively for the past three decades. Additionally, the versatility of aromatic thioketones has been explored extensively as building blocks for complex sulfur-containing compounds.

**Scheme 2:**
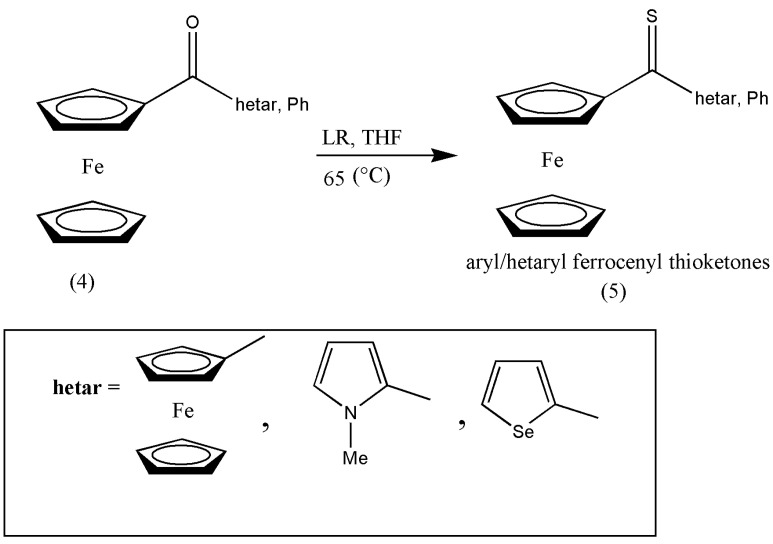
Synthesis of aryl/hetaryl ferrocenyl thioketones (5) via conventional methods.

Ferrocenyl-functionalized compounds have found a key place in materials chemistry, medicinal chemistry, polymer chemistry, and polymer sciences, but little is known about ferrocenyl-substituted thioketones despite their wide variety of applications. A series of ferrocenyl hetaryl ketones was prepared by G. Mloston et al. [18] by consequent thionation to provide thioketones (5). For the conversion of ferrocenyl hetar, phenyl ketone (4) was formulated by its reaction with LR in tetrahydrofuran (THF) at 65 °C. The impure product was purified via column chromatography in a 75–85% yield (Scheme 2).

The ferrocenyl and hetaryl thioketones are considered building blocks in the organic chemistry of sulfur. Two methods are formulated in the synthesis process via conventional and microwave-assisted techniques. First, the traditional practices are progressed in boiling THF or toluene, with low to high yields and prolonged reaction times of 1 to 2 h [18,19], which is overcome by applying microwave irradiation. The synthesized ferrocenyl thioketones are unique substrates to prepare the hitherto unknown ferrocenyl-substituted thiiranes and disparately substituted alkenes with diazo compounds [18].

Citing the requirement for hetaryl and ferrocenyl-functionalized thioketones in diverse synthetic applications such as synthesizing heterodienes via thia-Diels–Alder reactions and as reactive dipolarophiles in the [3+2]-cycloaddition with diazo compounds prompted Fałdyga et al. [20] to elaborate on efficient and practical methods for their synthesis.

The thioketones can be prepared via conventional and microwave irradiation (MW) methods, but the conventional techniques required prolonged reaction times of 1 to 2 h, and in some instances, the yield was not satisfactory. Therefore, MW techniques were explored to improve hitherto applied protocol.

**Scheme 3:**
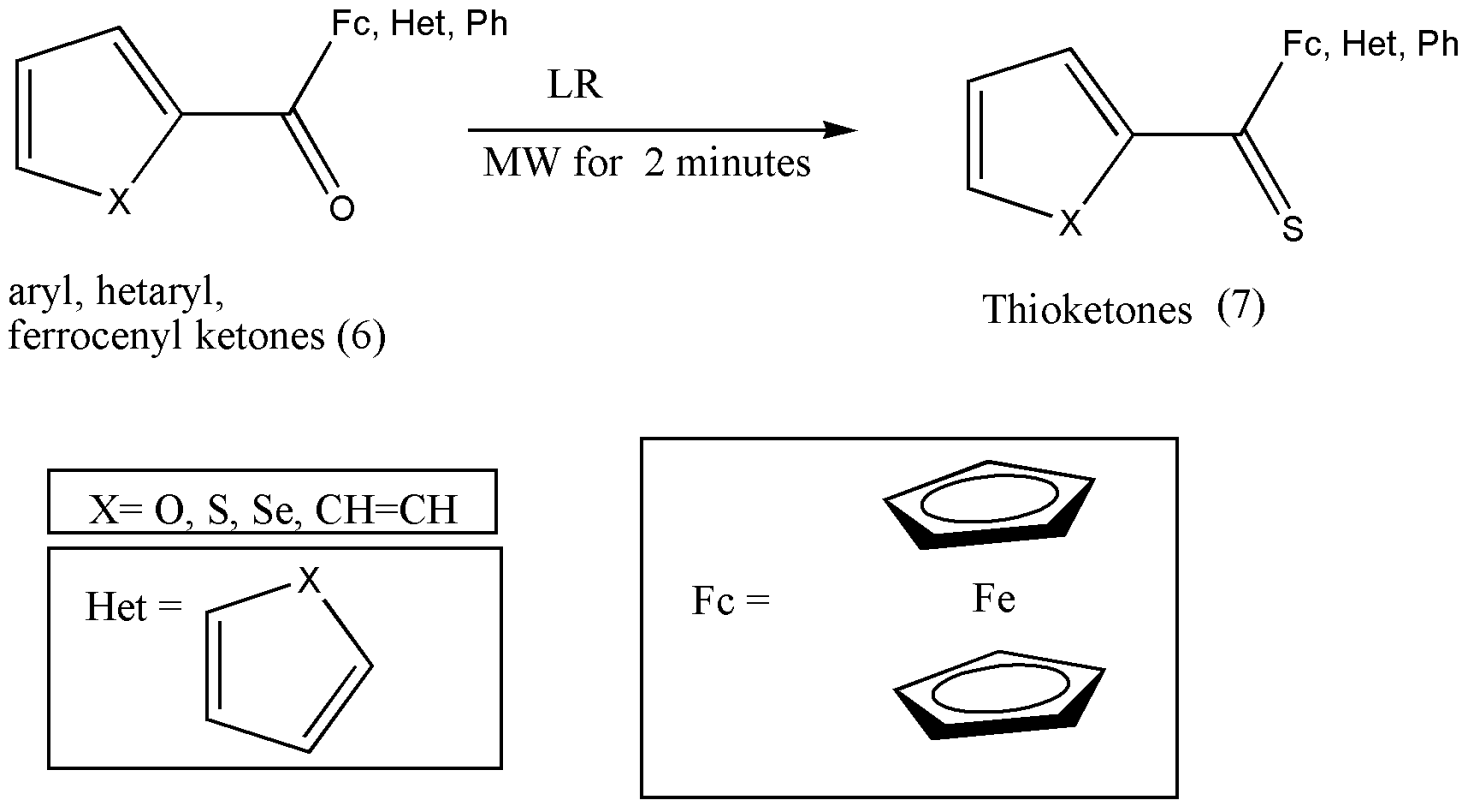
Microwave-assisted synthesis of thioketones (7).

Reactions were performed in toluene solution with LR and substituted ketones (6) placed in a reaction tube and irradiated for 2 min using 150 W of power. Further purification of the obtained compound (7) was achieved via flash chromatography using the eluents petroleum ether and dichloromethane (8:2) (Scheme 3) [20].

The synthesis of more diverse thioketones such as thiofluorenones should be explored and experimented with due to their importance in cycloaddition chemistry.

The most common approach employed for the synthesis of thioketones using LR involves reactants and a suitable solvent. The conventional methods usually demand an excess of LR and a longer reaction time of 2–25 h at higher temperatures in the presence of dry solvents. Notable work has been done to provide a high-yield synthetic pathway to transform ketones and other carbonyl compounds into their thio analogs [21] to avoid dry solvents and excess LRs.

Jasmine and jasmone analog odor fragrances are indispensable in the perfume industry. The conversion of jasmone to its thio analogs makes an exciting group of compounds, with profound possibilities, as S fragrant compounds have been widely registered. The significance of fragranced jasmone prompted Pawelczyk et al. [22] to synthesize thio analogs of jasmones under solvent-free conditions. Consequently, it has also been found that replacing conventional methods with microwave solvent-free methods for the thionation of jasmone heterocyclic analogs to thio analogs have been observed in quite a bit of research literature due to its high-yielding, environmentally friendly, and economical procedures [21,23,24].

**Scheme 4:**
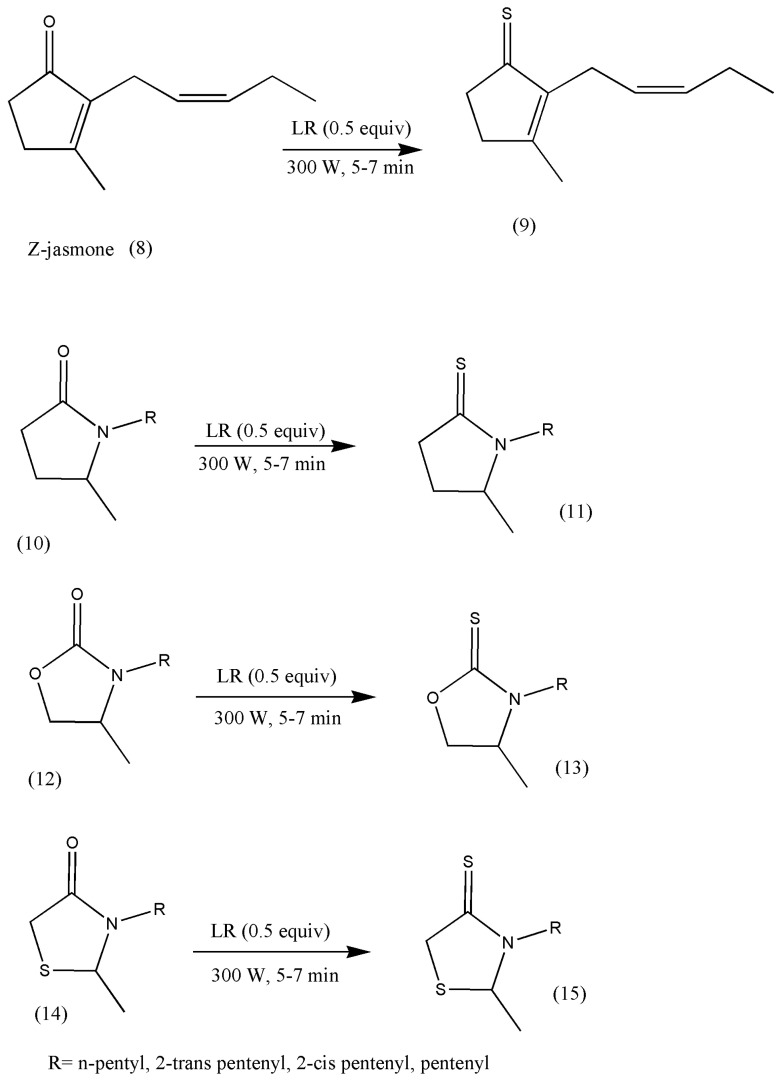
Synthetic pathways to prepare fragrant thioketones (9, 11, 13, 15).

These new jasmine analogs were centered on five-membered heterocyclic ketones containing one or two heteroatoms, for example, oxazolidione, pyrrolidinone, thiazolidinone, and Z-jasmone. The reactions progressed in microwave conditions to reduce time to just a few minutes compared to conventional methods (Scheme 4).

A survey of the olfactory properties of the synthesized compounds signaled that thioheterocyclic analogs of jasmone demonstrate a substantial variety of odors but exhibit relatively less advantageous odor properties than oxygen analogs because all thionated products lack the jasmine character.

However, their odors are intensive, such as fruity, floral, vegetable, or herbal, with impressive durability, similar to those of jasmone heteroanalogs. Their odors are unpleasant and spicy notes at first, which become better over time. These new synthetic odor notes have found a place in perfumery, flavor, and fragrance industries [24]. Further research in this field is imperative to make the perfume industry flourish.

Another class of pharmacologically essential thioketones is derivatives of quinazoline-thiones, which has shown a growth in pharmacological activities on the conversion of quinazolin-4-one to its thiones [25,26]. The intended conversions and the pharmacological investigations were conducted in achiral or racemic quinazolinone substrates.

The thione derivatives of quinazolin-4-ones are generally prepared by using P_2_S_5_ as a thionating reagent in low yields. However, this thionation requires long reaction times (12 h) under xylene reflux conditions [26].

**Scheme 5:**
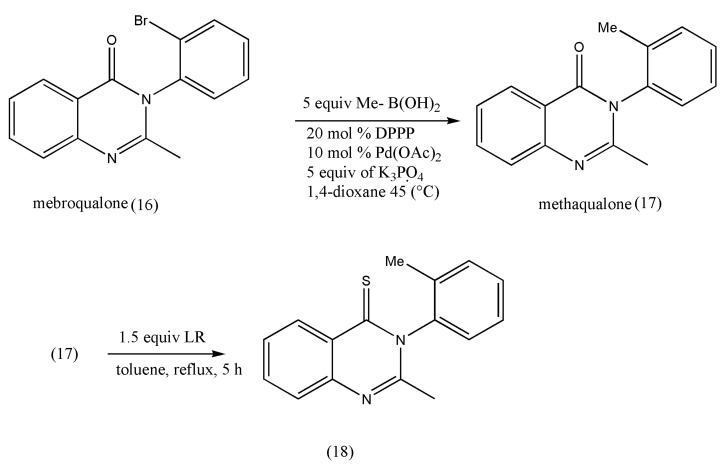
Synthetic pathway for the thionation of mebroqualone (16) to afford (18).

Under these extreme conditions, quinazolinone’s thionation also decreases optical purity due to rotation around a chiral axis. The above drawbacks were overcome using LR, which is more reactive than P_2_S_5_, as demonstrated by Niijima et al. [27]. Under xylene reflux conditions, the mebroqualone (16) reaction with 1.0 equiv of LR was completed in 3 h to give thioketones in 87% yields, but a decrease in enantiomeric excess (ee; 94%) was observed. The conversion of mebroqualone to methaqualone (17) via Suzuki–Miyaura coupling increased the ee to 99%. These findings indicate that the ee of thionation products is greatly influenced by ortho-substituents (Scheme 5).

**Scheme 6:**
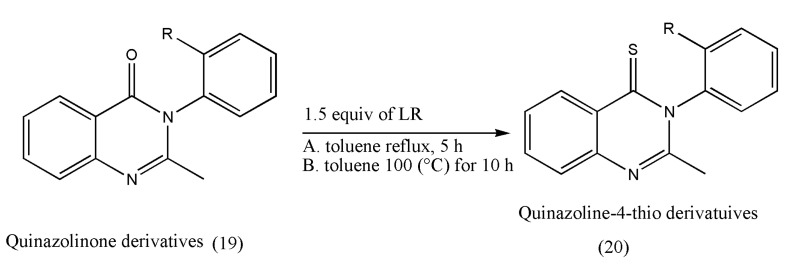
Synthetic pathways to prepare quinazoline-4-thio derivatives (20).

Following the success of the thionation of mebroqualone, the reactions proceeded with optically pure quinazolinones bearing various substitutions at ortho positions. The reactions of optically pure quinazolinones (entry 1–7) with different ortho-substituted phenyl groups (Scheme 6) have been summarized in Table 1.

As seen in Table 1, ortho-ethyl and ortho-isopropyl derivatives show an ee of 99% compared to ortho-methyl derivatives. These results point out that the magnitude of decrease in the ee is due to the increase in the ortho-substituents’ steric bulkiness. A similar tendency was also seen in the thionation of ortho-halo derivatives. The ortho-chloro derivatives displayed decreased ee (95%), whereas ortho-iodo derivatives progressed without reducing ee (99%).

Scheme 6 is the first example of the thionation of optically active N−C axially chiral amide compounds and offers new ways to synthesize pharmaceutically active quinazoline-4-thione derivatives.

## 3. Synthesis of Biologically Active Thioamides

Thioamides were first prepared in the year 1815 by Gay-Lussak and later by Berzelius in 1843. The conversion of oxygen of amides to its thio analogs is the basis of thioamide formations.

Even though they are structurally pretty similar to each other, there is a vast difference in the chemical properties of these two species. Thioamides are more polar than their respective amides, making them participate in more reactions. The chemical adaptability of thioamides makes them an excellent molecule for their extensive studies. The resourcefulness of thioamides makes them a good starting material to prepare thiazoles, amidenes, amidrazones, and many more [28].

The structural significance of thioamides has led researchers to investigate their biological properties [28,29].

**Scheme 7:**
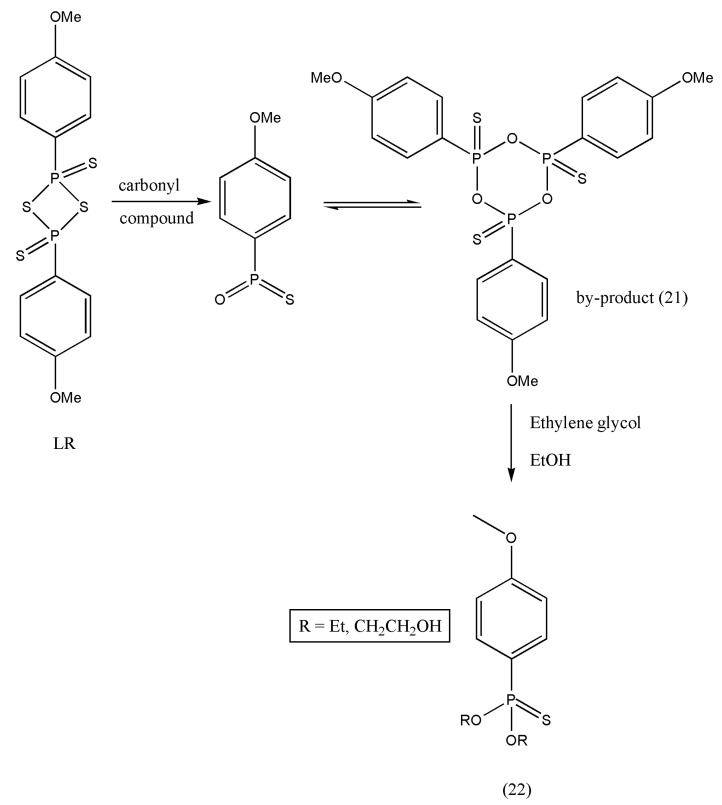
Formation of six-membered by-product (21).

Thioamides’ synthesis appeals to organic chemists because of their applications in various fields such as pharmaceuticals, agrochemicals, electronic chemicals, and material science [30,31,32,33,34,35]. Moreover, the thio-substitution of the amide with LR is a simple, efficient, and straightforward reaction. However, the work-up procedure is constantly under criticism despite LR being a powerful, mild, and flexible thionating agent [36,37]. A six-membered ring structure (21) is formed as LR completes thio-substitution [38]. However, compound (21) polarity is similar to the desired products, and the purification of selected products becomes tedious and less efficient because of its good solubility; concerning this, LR is always limited to a small-scale preparation. An efficient work-up procedure decomposing by-products (21) of LR in ethylene glycol (Scheme 7) was formulated by Ke Wu et al. [39]. The freshly designed, column-free procedure avoided P-contained aqueous waste, and only organic effluents were released. The optimized approach was articulated to offer an opportunity to apply the LR for numerous thio-substituted reactions in scaling up preparations.

**Scheme 8:**
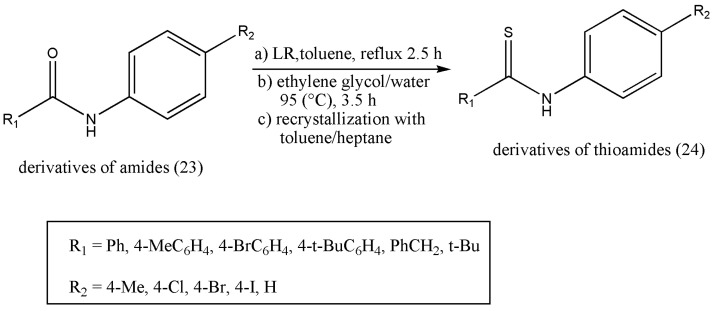
Synthesis of thioamides (24).

The exemplified reaction pathway of Scheme 8 demonstrates the reaction of amide derivatives (0.20 mol) (23) and LR (0.102 mol). An excess of ethylene glycol decomposed the by-product A.

In the above example of thioamides, ethylene glycol treatment allowed the work-up process comprising phase-cut, back extraction, activated carbon treatment, and re-crystallization in an appropriate solvent. As a result, some of the solvents were re-used, and effluents were reduced. This method can be used in scaling preparation with the application of Lawesson’s reagent.

Thioamides have a more decisive dipole moment than their analogous amides, and if the nitrogen remains unsubstituted in an amino group, they are better hydrogen bond donors. The antimycobacterial properties of thioamides have been investigated, and these functional groups are present in novel antibiotics such as sulfinemycin and chlosthioamide, and the former is related to the antitubercular compound 2-(decylsulfonyl)acetamide (25). Hsien-Kuo Sun et al. [29] reasoned that introducing the thioamide group could improve the potent antitubercular activity of the compound (25).

The compound 2-(decylsulfonyl)acetamide (0.46 mmol) (25) was converted to thioamide (26) by dissolving in 20 mL toluene and LR (0.628 mmol).

**Scheme 9:**
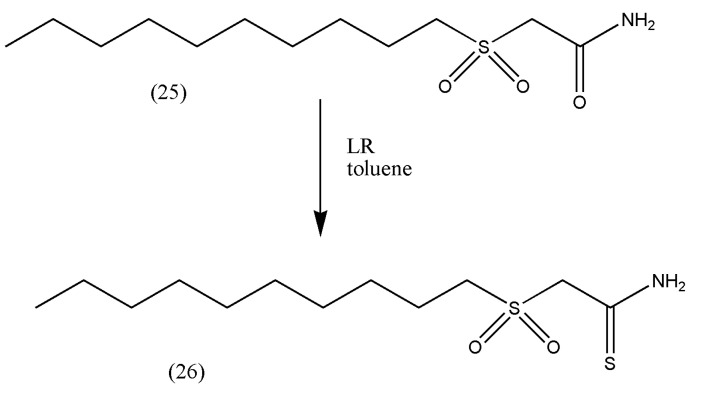
Synthesis of thioamides (26).

The reaction progressed for 2 h, and the excess solvent was removed via vacuum (Scheme 9).

The thioamide (26) showed slightly better activity against *Staphylococcus aureus* than tuberculosis, which is remarkable owing to the simplicity of the compounds’ structure. However, further studies on these compounds should be enhanced due to their great activity against the second-most major pathogen.

Aromatic oligoamides are defined as shape-persistent, uncollapsable structural molecules due to their intramolecular hydrogen bonding.

The introduction of soft donor atoms such as sulfur into amide carbonyl oxygen of oligoamides can improve complexation properties. In recent years, the selective recognition of transition metal ions has been a subject of interest due to the need for selective and efficient detection of biologically essential cations (e.g., Cu^2+^, Fe^3+^, Cr^3+^, Pb^2+^, Hg^2+^). The selective receptors for the soft transition metal copper ions have been researched using shape-persistent aromatic oligothioamides containing hydrophobic lumen devised by Qian Jiang et al. [40]. These unique aromatic oligothioamides have a selective recognition for copper(II) ions.

**Scheme 10:**
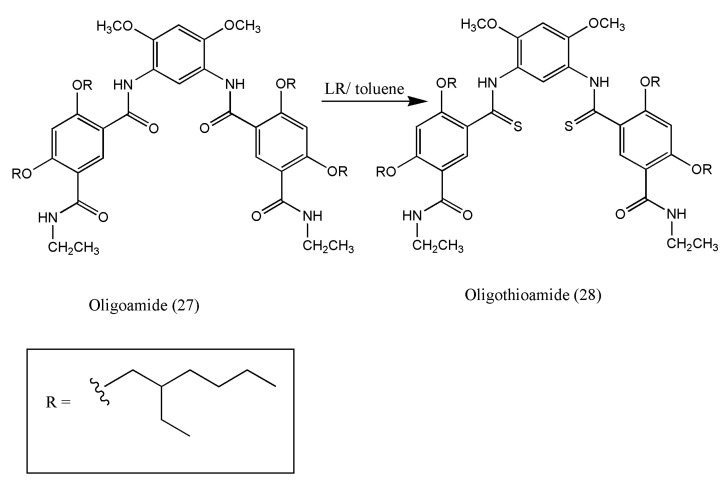
Synthetic pathways to prepare oligothioamides (28).

To a solution of oligoamides (0.78 mmol) (27) in dry toluene (20 mL), LR (1.86 mmol) was added to give oligothioamides (28) (Scheme 10).

Further investigation of oligothioamides via UV–vis spectra proved to have high selectivity for Cu^2+^ ions due to the presence of highly electron-rich S atoms. Therefore, further studies in these fields are imminent and should be researched for other biologically important soft cations.

The natural amino acid L-proline has been portrayed to catalyze numerous (more than 10) enantioselective C–C and C–heteroatom bond-developing reactions. L-prolines are considered as an advantaged catalyst and have inhabited a central role. Even though they can effectively catalyze countless asymmetric transformations, certain limitations need to be addressed, such as minimal miscibility in organic solvents, high catalyst loadings, and laborious tuning of its reactivity through structural changes. Many derivatives based on the proline framework have been synthesized, and their catalytic properties have been assessed [41,42,43] with enhanced reactivity and selectivity. Further advancement in this field was brought about by Wang et al. [44]. The authors combined a dipeptide functional group with the proline thioamide forming thioamide-type dipeptide derivative built on the proline catalysis concept and double hydrogen bonding activation and inspected on organometallic asymmetric aldol condensation.

**Scheme 11:**
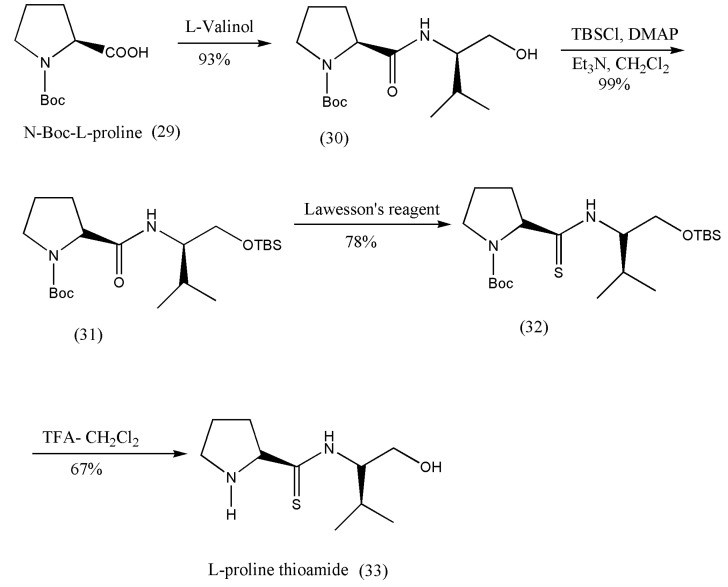
Synthetic pathway to prepare L-proline thioamide (33).

A derivative (33) was obtained from N-Boc-L-proline, reacted with the apposite L-valinol, then protected hydroxyl group with TBSCl, thiation with LR, and deprotection of the amino and hydroxyl groups (Scheme 11).

Application of proline thioamide as a catalyst in direct aldol condensations was examined with numerous aldehydes, including aromatic and aliphatic aldehydes. The aromatic aldehydes bearing an electron-withdrawing group offered good conversions, whereas less electrophilic aldehydes gave the aldol products low yields. On the other hand, the aliphatic aldehydes proved ineffective for aldol condensation because of meager results [44]. The terminal hydroxyl group of the catalyst proline thioamide is a primary alcohol, different from previously reported secondary or tertiary alcohols. The designed catalyst plays a crucial role in asymmetric aldol condensation and should be taken as a hint to modify this organocatalyst further.

## 4. Synthesis of Biologically Active Thioesters and Thionolactones

Swapping one or more oxygen atoms of esters and lactones with a sulfur atom using LR has been displayed with various examples below; although the thionation of ester functional groups has been reported to be complicated [45]. In addition, the thionation of esters requires prolonged reaction time, with refluxing in usual LR solvents such as toluene and xylene. However, employing the microwave method can shorten the reaction time to a few minutes [21].

**Scheme 12:**
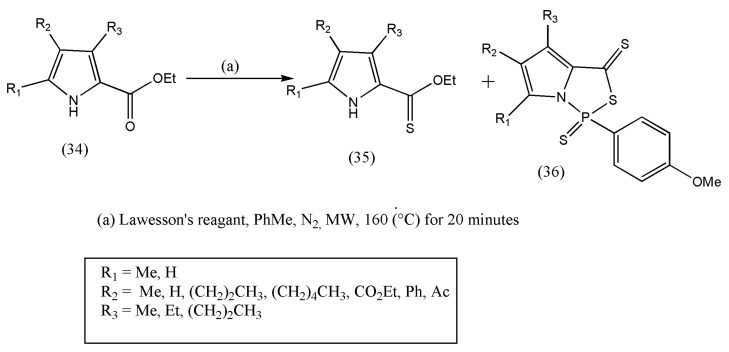
Synthetic pathway to prepare thionoester (35) and (1,3,2)-thiazaphospholidine (36).

Pyrrole-containing sulfur functionalities are scarce. Thioester-containing pyrroles are best known for their vital role as thioesterase-appended pyrroles in the biosynthesis of secondary metabolites [46,47]. Literature review of pyrroles affixed with thionoesters is constricted to mono and di-pyrroles reacting with dithiocarbonates and methanolic solutions of thiophosgene [48,49]. With the knowledge of thioesters and to overcome the limitations, Groves et al. [50] synthesized thionoesters with 2-pyrrole carboxylate and Lawesson’s reagent at higher temperatures. A new class of pyrroles annulated with (1,3,2)-thiazaphospholidine unit were also reported (Scheme 12).

The synthetic work started with ethyl-2-pyrrole carboxylate, which is commercially available via Knorr-type condensation. Next, the microwave-assisted technique was applied instead of conventional methods, which endured long reaction times using LR.

The total material consumption (34) was accompanied by two new compounds (35, 36). The compounds were identified, and the desired product (35) was isolated with a 59% yield. Even though the (1,3,2)-thiazaphosphole functional group (N–P–S) is well defined, the authors could not find pyrrolic examples displaying this functionality [50]. On the other hand, the four-, five-, and six-membered cyclic thiazaphospholes were reported to be well defined. There is a possibility of adverse interactions between LR and 2-pyrrole carboxylates and the formation of dipyrins and their complexation products. This issue needs to be considered, and researchers should make further modifications.

To conclude, the authors successfully prepared pyrrole thioesters in moderate to high yields with mild reaction conditions, and this method should be extensively studied to synthesize new classes of thioesters with pronounced biological activities for advancement in medicinal chemistry.

Another research example of pyrrole subunit in porphyrin analogs is porpholactones porphyrins in which one porphyrin β,β′ double bond was substituted by a lactone moiety. The availability of porpholactones and the periphery functionality of the macrocycle that is electronically combined with the chromophore makes them an enticing study material. The conversion of porpholactone to porphothiolactones affects the structure and electronic properties of the chromophore.

**Scheme 13:**
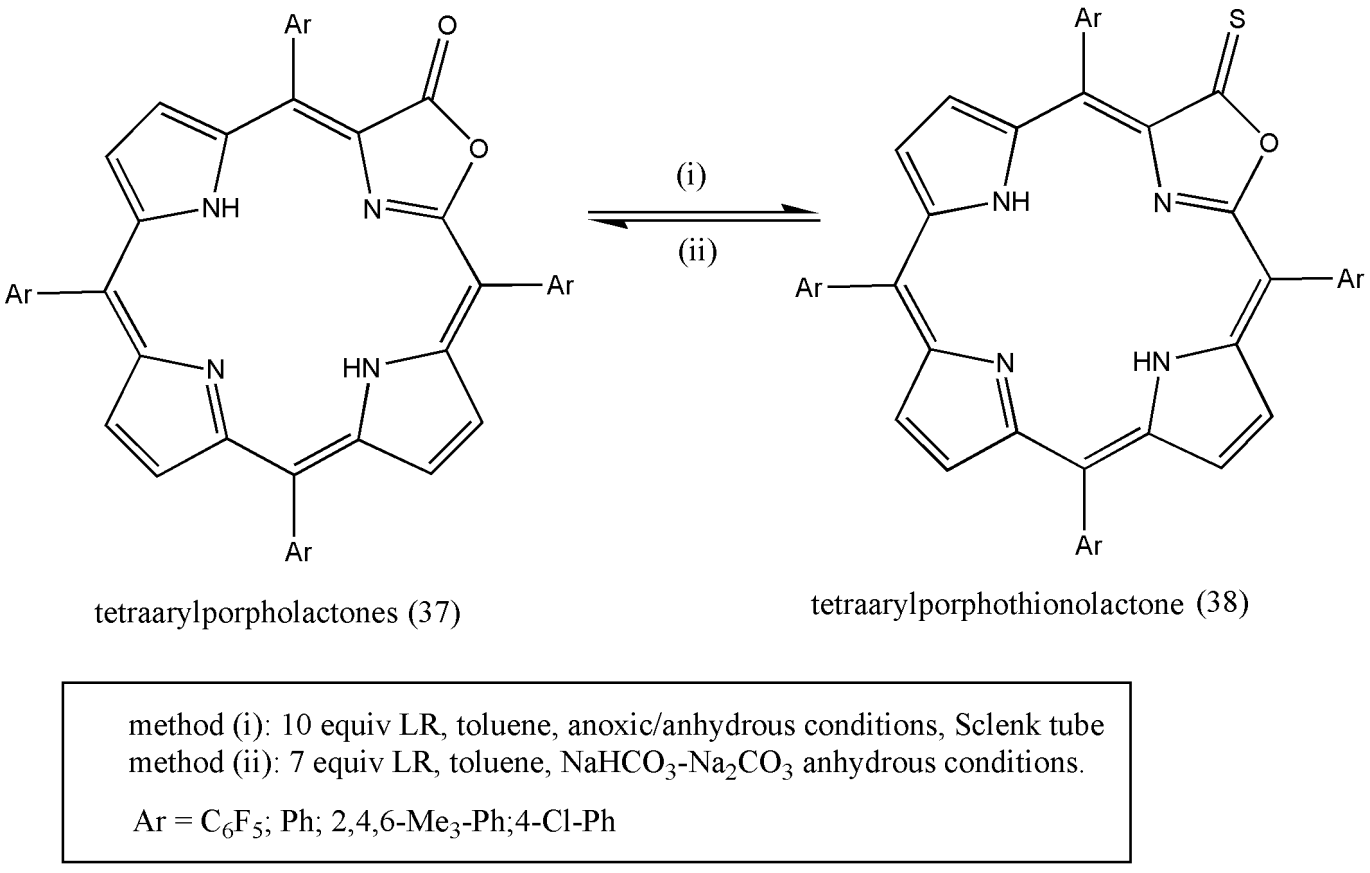
Synthetic pathway to prepare porphothionolactone (38).

The exchange of lactone moiety to a thionolactone using Lawesson’s reagent has been studied and synthesized by Yi Yu et al. [51]. The reaction of porpholactones (37) with an excess of LR yields good to excellent results (Scheme 13). In addition, the presence of a base in the reaction mixture makes the reaction more enhanced. As a result, the product thionolactone (38) was formed, and analytical data indicated that only ketone–thione conversion had occurred.

Additionally, thionolactones can be used to prepare oxazolochlorins using RANEY^®^ nickel-induced hydrodesulfurization reactions. Some thionolactones have also proved to be disinfectants and have biological significance in the inflammatory process [51]. The synthesized thionolactones can be utilized as a starting mode for further modifications.

Fertilizers and pesticides play a vital role in achieving a higher yield in agricultural production and minimizing the overall loss of productivity caused by harmful agents. However, the intensive and indiscriminate use of these products is a cause of environmental pollution and can be detrimental to human health. Therefore, researchers continuously have to develop new classes of synthetic molecules in conjunction with safer toxicological and environmental profiles. It is well noted that almost 30% of agrochemicals have at least one sulfur atom, mainly in insecticides, herbicides, and fungicides.

The biological significance of sulfurated heterocyclic derivatives with promising herbicidal properties was further researched by Salvatore et al. [52].

**Scheme 14:**
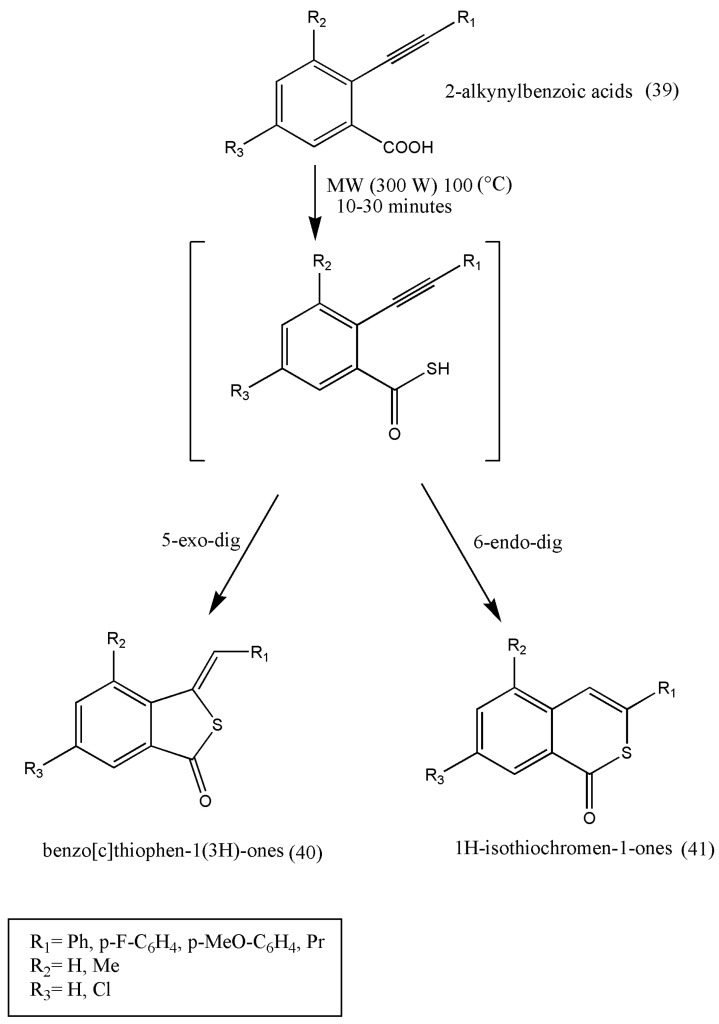
Synthetic pathway to prepare sulfurated heterocycles (40, 41).

This approach is based on the tandem thionation of 2-alkynylbenzoic acids in the presence of LR under microwave irradiation. It was found that 0.5 equiv of LR, with 2-alkynylbenzoic acid (39), after a short reaction time of 10–30 min, it is possible to change (39) into benzo[c]thiophen-1(3H)-one (40) or 1H-isothiochromen-1-one (41). These products are derived from 5-exo-dig or 6-endo-dig cycloisomerization of 2-ethynylbenzothioic acids (Scheme 14). The sulfurated heterocycles obtained were tested as herbicides in agriculture.

Further studies should be evaluated on crops and weeds and should be assayed on their potential danger to human and animal health.

## 5. Synthesis of Thioacids

Thioacid represents a firm and resourceful class of organic compounds, and due to their nucleophilicity, thioacids have found the synthesis of thiols and thioesters [44] and in azides and nitrosulfonamides [53,54]. They can also function as acylating agents under mild conditions, which has led to the development of simple amides and amide bonds in peptide ligations [55].

Rao et al. [56] have outlined the direct conversion of the carboxylic acid with LR. The authors postulated that the carboxylic acid would react with LR as observed in alcohols through a “Wittig-like” mechanism [57].

**Scheme 15:**
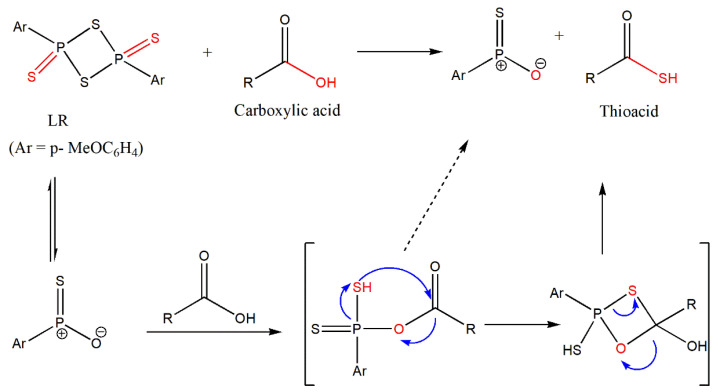
The proposed mechanism of thioacids formation with LR.

The outlined Scheme 15 postulates the direct reaction of a carboxylic acid with LR analogous to alcohols, similar to the Wittig mechanism to offer thioacids [56].

Hence, the initial reaction was carried out using benzoic acid, followed by other examples.

**Scheme 16:**
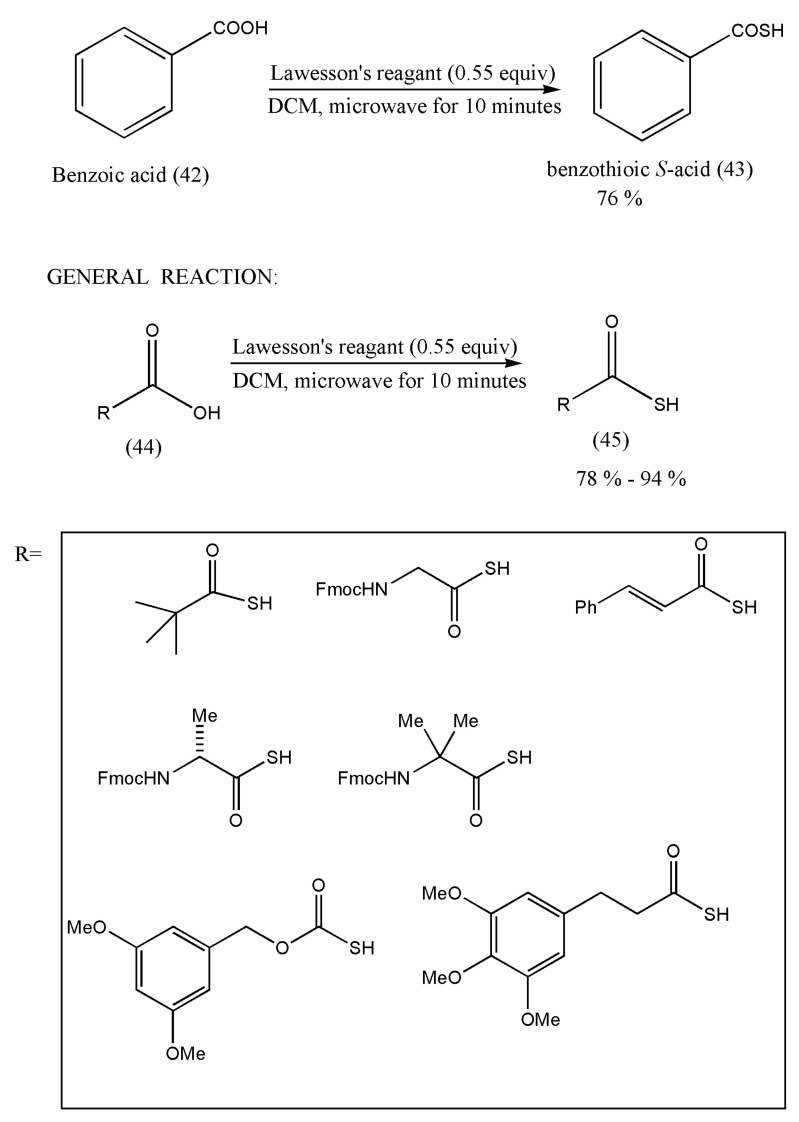
Synthetic pathway to prepare thioacids (43, 45).

The general method to prepare thioacids using LR has been depicted in Scheme 16. The commercially available carboxylic acid (42, 44) (0.50 mmol) and LR (0.28 mmol) in DCM (2.0 mL) were exposed to microwave irradiation for 10 min at 100 (°C). The final purification of thioacids (43, 45) was executed by flash column chromatography.

Therefore, the authors were successful in synthesizing aliphatic thioacids and also hindered thioacids in good yields. However, substrates with more reactive alcohols and primary amide functional groups did not produce the desired products. A good reason for this may be attributed to the carboxylic acid group’s competition with alcohols and amides with LR. Furthermore, this synthesis method should be explored with a different type of carboxylic acid.

Since this is the first direct conversion method of carboxylic acid to thioacids, further study on the synthesis of more thioacids should be researched. Furthermore, their biological applications should also be taken into consideration.

## 6. Synthesis of Biologically Active Thiols and Thioethers

Thiols and thioethers are a vital class of organic compounds with pronounced importance for organic synthesis. Various methods are well known for their preparation [58,59], and in the latest years, asymmetric synthesis of thiols has been researched significantly [60]. Furthermore, it is known that several thiols and thioethers [59] are biologically active substances [61], and some of them are of particular interest as odorous compounds [62].

Aryl sulfides are essential molecules in various anti-HIV, Alzheimer’s disease, and diabetes medications. According to the literature review, previous preparation methods of aryl sulfides, such as Ullmann-type reaction of arene thiols with aryl halides or Grignard reagants with sulfur-based electrophiles, which proceed under harsh reaction conditions, render them suitable [63,64]. The LR as a sulfur surrogate and copper(I) iodide as a catalyst in diglyme has been described as a new method for the thioetherification of alcohols by Mohammad Gholinejad [63].

**Scheme 17:**
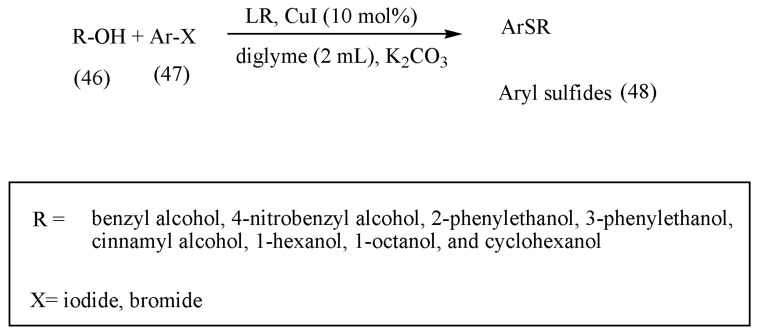
Synthetic pathway to prepare thioethers (48).

Noting the importance of LR in thionation reaction, the author initially tested the feasibility of the reaction with aryl iodides, structurally different alcohols, and LR with CuI as a catalyst (Scheme 17). The reaction progressed well with high to excellent yields. Furthermore, the reaction was also tested with different aryl halides such as aryl bromides, which also afforded sulfides in high yields.

The significance of the above reaction lies in the use of numerous commercially available alcohols affording thioethers with the same high yields. Besides, a one-pot synthesis of thioethers was developed using mild conditions.

Fluorinated alcohols and thiols are essential building blocks in fluorine medicinal chemistry, crop protection, and material science. Even though perfluorinated alcohols are of great interest in material chemistry, synthesizing trifluoromethyl thiols is reported scarcely. The tertiary trifluoromethyl thiols were synthesized via aromatic thioketones and Ruppert–Prakash reagent, but a mixture of products was formed, leading to the low yield of thiols [65].

The unsuccessful attempt to prepare trifluoromethyl thiols inspired Mloston et al. [65] to examine selected alcohols with LR. The powerful thionating properties of LR are well known, as well as the conversion of alcohols to thiols.

**Scheme 18:**
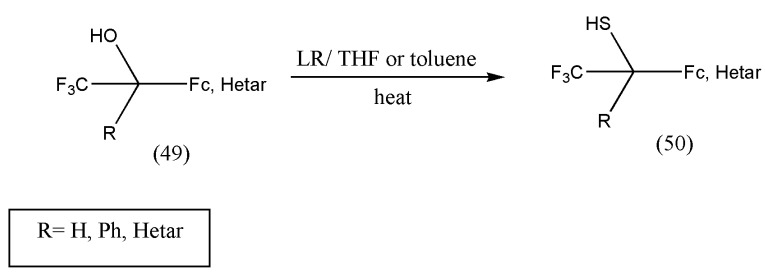
Synthetic pathway to prepare trifluoro thiols (50).

The heating of alcohols (49) in THF or toluene in the presence of slight excess of LR resulted in the synthesis of thiols (50) in moderate yields (Scheme 18).

The prepared fluorinated alcohols and thiols are new products and essential for designing more complex fluorinated compounds. An essential feature of fluorinated thiols was that they did not display an unpleasant odor of alkanethiols.

**Scheme 19:**
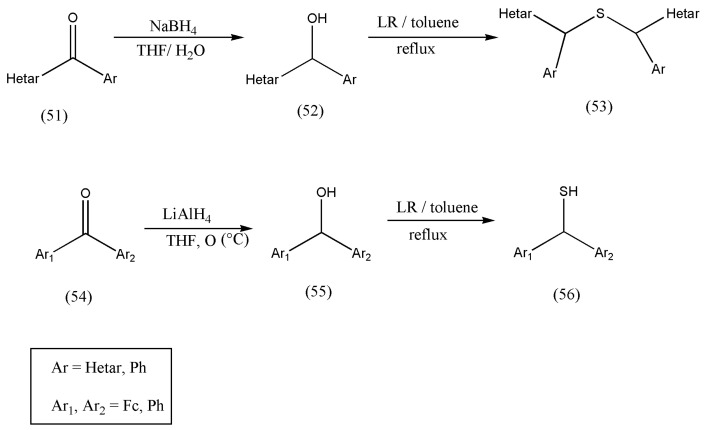
Synthetic pathways to prepare disubstituted methanols and their transformations into sulfides (53) or thiols (56).

After a successful attempt to prepare primary, secondary, or tertiary alcohols with LR to their corresponding thiols, the authors Mloston et al. [66] synthesized a new series of thiols from hetaryl and ferrocenyl-substituted ketones (51, 54). The hetaryl and ferrocenyl-substituted ketones were converted into secondary alcohols via reduction with NaBH_4_ in THF solution and lithium aluminum hydride, in the case of phenyl/ferrocenyl ketones. Scheme 19 exemplifies the synthesis of thiols using secondary alcohols (52, 55). The ketones (51) were transformed to alcohols via reduction with NaBH_4_ in THF solution, and lithium aluminum hydride (LiAlH_4_) was used in 54.

**Scheme 20:**
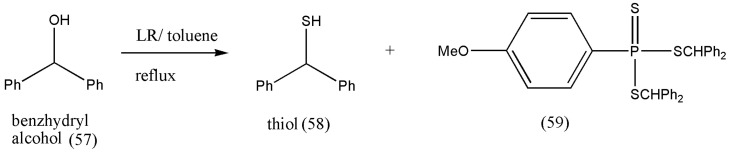
Synthetic pathway to prepare thiols (58) from benzhydryl alcohol (57).

According to the literature review, the reaction of benzhydryl alcohol with LR leads to a side product of the type bis(diphenylmethyl)phenyltrithio-phosphonate (59) [67] (Scheme 20). However, the formation of (59) was not observed in wet toluene. Thiol (58) was formed as the only product with a 90% yield [66].

Therefore, all products obtained from secondary alcohols drawn from methanol strongly depend on the substitution pattern of hetaryl and ferrocenyl groups and are significant.

Therefore, the above reaction (Scheme 19 and Scheme 20) emphasizes the importance of the hetaryl group because both benzhydryl alcohol and ferrocenyl(phenyl) methanol behaved analogously by forming only respective thiols. To conclude, the type of thiol formed from secondary alcohols derived from methanols is based on the substitution pattern of hetaryl and ferrocenyl groups.

To conclude, these results point out that the presence of the hetaryl group strongly modifies the nucleophilicity of the SH group, which in turn modifies its reactivity. Thiol is a necessary reagent for synthesizing sulfides and disulfides, which are essential molecules in drugs related to sulfur chemistry.

Shikonin derivatives are potent anticancer molecules and have gained considerable attention in recent years [68].

Incorporating a sulfur group into the stereogenic carbon of the shikonin derivative has not been reported.

Therefore, a class of thiols (R or S)-1-(1,4,5,8-tetramethoxynaphthalen-2-yl)pent-3-ene-1-thiol (61) as sulfur-containing shikonin derivatives was initiated by Guang Huang et al. [69], which acted as the key intermediate in the synthesis of bioactive molecules.

**Scheme 21:**
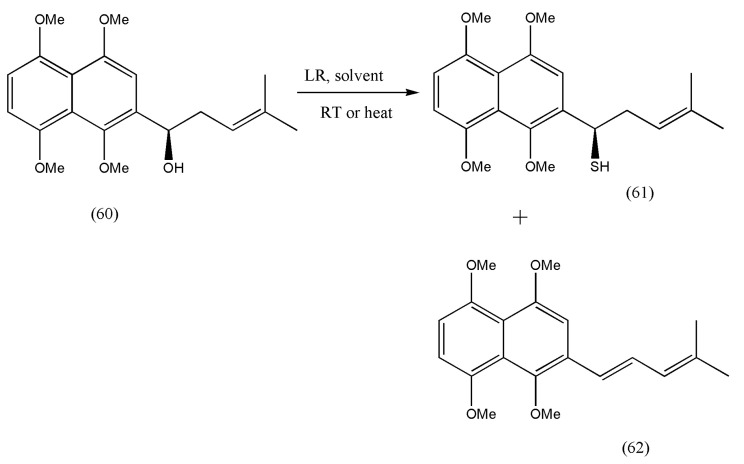
Synthesis of thiols (61).

As depicted in Scheme 21, the reaction progressed on reaction (60) with LR to afford (61), but a dehydration product of alcohol was also formed (62).

The optimization of reaction conditions was studied in Table 2.

As per the reaction conditions of Table 2, it was observed that the products formed were low in DME, CH_2_Cl_2_, 1,4-dioxane, and CH_3_CN at room temperature, but an increase in yields was obtained when alcohol (61) was heated with LR in CH_3_CN or toluene. In addition, an increase in the output of dehydration products (62) was observed due to excess heating. Therefore, the optimum reaction conditions were fixed at entry no. 13.

Therefore, this method can be a perfect example to prepare a multigram-scale synthesis of chiral thiols.

## 7. Thiadiazoles

**Figure 2:**
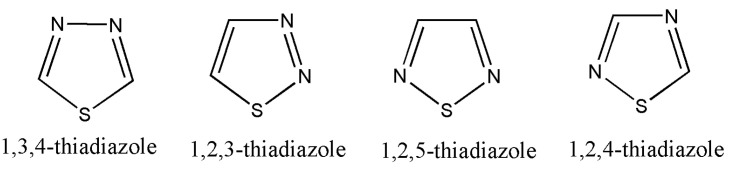
Isomers of thiadiazoles.

Thiadiazoles are five-membered heterocyclic compounds containing one sulfur atom and two nitrogen atoms and are numbered, as shown in Figure 2 [70]:

Of all the derivatives of thiadiazoles, 1,3,4-thiadiazole are commonly synthesized and widely studied. Other derivatives such as 1,2,4/1,2,3/1,2,5 have a minimum information available on literature review.

The benchmark method to prepare 1,3,4-thiadiazoles comprises cyclization of acyl hydrazines or thiohydrazines, usually employing dehydrating agents such as POCl_3_ [71,72], PCl_5_ [73], FeCl_3_ [74], or H_2_SO_4_ [75].

Thionation of acyl hydrazines with LR followed by cyclization and dehydrosulfurization has been reported to afford 1,3,4-thiadiazoles in good yields. The application of microwave irradiation in chemical reactions has gained momentum recently due to improved reaction rates and yields.

In continuation of the research to synthesize new 1,3,4-thiadiazoles derivatives under microwave conditions, a series of 1,3,4-thiadiazole derivatives, including 5-phenyl-2- furan moiety, were synthesized by Zi-Ning Cu et al. [76] and were found to have pronounced biological activities. Furthermore, intermediate 5-substituted phenyl-2-furoic acids (65) were prepared from aniline derivatives (63) and 2-furoic acids by Meerwein arylation reaction [76].

**Scheme 22:**
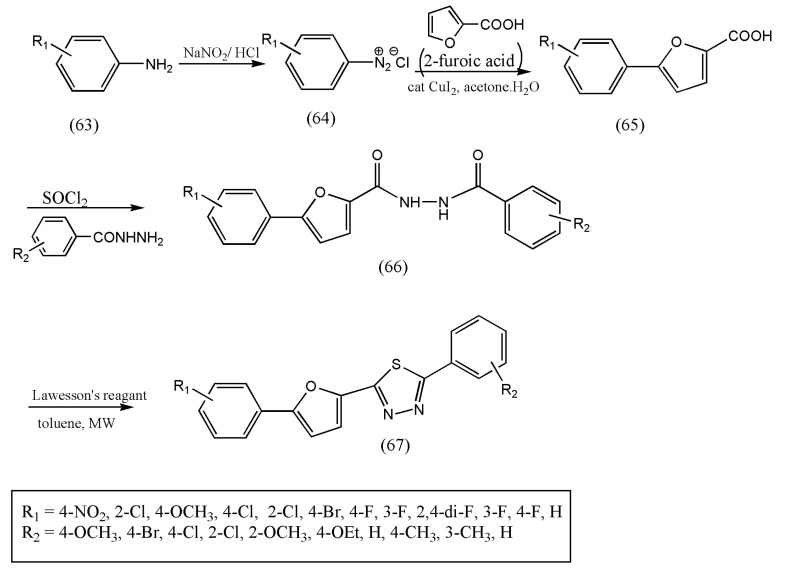
The synthetic pathway to prepare substituted 1,3,4-thiadiazole (67).

The thionation of diacylhydrazines (66) with LR followed by oxidative cyclization in dry toluene formed the title compounds 1,3,4-thiadiazoles (67). The authors proved this method economical and swift, and the reaction was completed in 15 min via microwave irradiation (Scheme 22). The reaction of diacylhydrazines with LR was tested with different solvents such as THF, dioxane, and xylene but provided low yields than toluene.

All the synthesized compounds displayed significant fungicidal properties against diverse fungi, especially against *P. infestans*. Therefore, these results indicate that the title compounds are promising lead compounds for further discovery, especially against *P. infastans*. Further research on the mode of action of title compounds should be investigated.

The biological diversity of 1,3,4-thiadiazole is well known and is found in commercially available drugs containing the 1,3,4-thiadiazole rings, such as megazol, acetazolamide (diamox), furidiazine, and desaglybuzole [77].

Since most of the methods reported to synthesize 1,3,4-thiadiazoles require harsh conditions and multi-step synthesis and have difficulty in forming non-symmetric thiadiazoles, there was a necessity to devise one-pot synthetic strategies. Therefore, Inseok Ko et al. [77] articulated a one-pot synthetic plan to prepare 2,5-disubstituted-1,3,4-thiadiazoles from aldehydes and hydrazides utilizing a series of N-aroylhydrazone formation, thionation, cyclization, and oxidation.

**Scheme 23:**
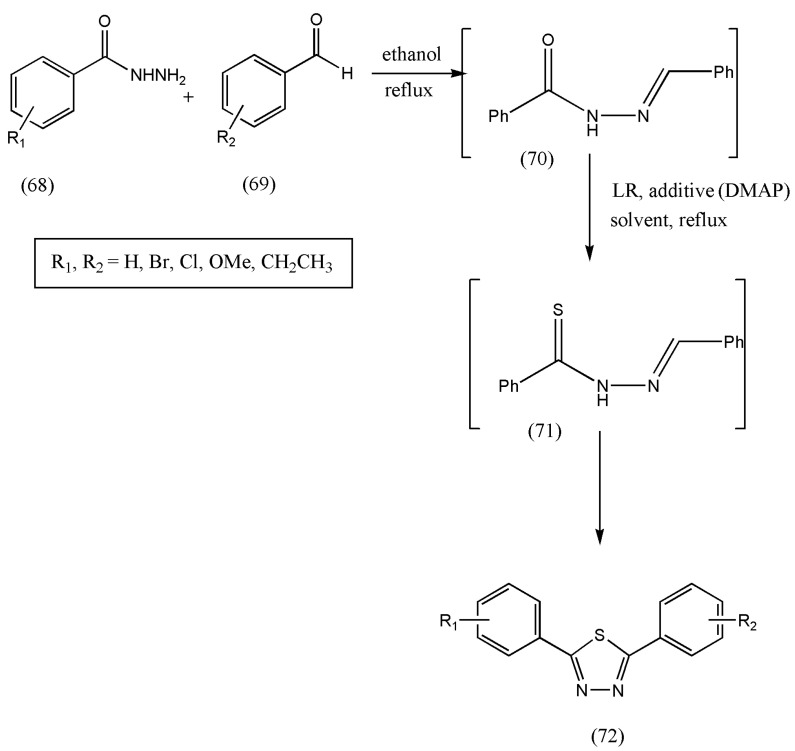
Synthetic pathway to prepare 2,5-diphenyl-1,3,4-thiadiazole derivatives (72).

As depicted in Scheme 23, the reaction of benzoylhydrazide (68) with benzaldehyde (69) leads to N-benzoyl hydrazone (70) formation without separation. When compound (70) was treated with LR, the disubstituted-1,3,4-thiadiazole (72) was yielded without oxidant, and thiohydrazide (71) was separated. The reaction progressed for 2 h in the first step, and excess solvent was removed via vacuum. Furthermore, to the remaining crude product (70), LR was added, followed by adding a base and refluxing for 10 h. The final purification of the crude product was conducted by flash column chromatography.

According to initial biological experiments by the authors, some of the newly synthesized thiadiazoles show antioxidant activity. Further investigations with results should be performed on the newly synthesized compounds for antioxidant properties.

**Figure 3:**
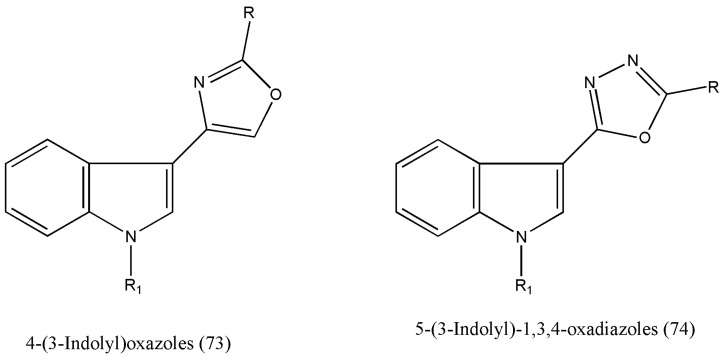
Structures of indolyl-based heterocycles.

Many natural and synthetic indole-based heterocycles with diverse mechanisms of action have been studied as anticancer molecules [78,79]. The authors Kumar et al. [80] have reported the synthesis of 4-(3-indolyl)oxazoles (73) and 5-(3-indolyl)-1,3,4-oxadiazoles (74) as potential anticancer agents against many types of human cancer cell lines (Figure 3) [80].

The authors extended their work on the new class of 5-(3-indolyl)-2-substituted-1,3,4-thiadiazoles owing to their anticancer properties. Encouraging biological activities of indolylzoles prompted the authors to alter the five-membered heterocyclic ring and indolyl moiety further to boost the structure–activity relationship (SAR), leading to potent and selective anticancer agents.

**Scheme 24:**
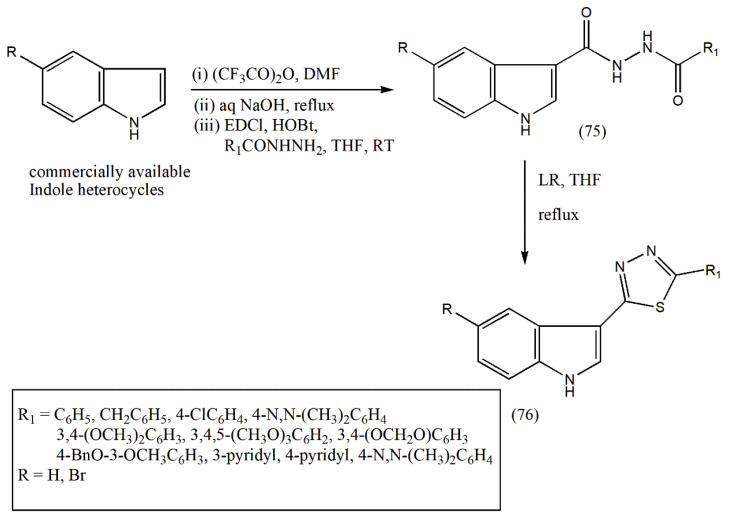
Synthetic pathway to prepare 5-(3-indolyl)-2-substituted-1,3,4-thiadiazoles (76).

The synthesis of 5-(3-indolyl)thiadiazoles (76) was attained via a three-step procedure from a commercially available indole, as outlined in Scheme 24. First, the commercially available indoles were treated with trifluoroacetic anhydride, followed by hydrolysis with NaOH. Then, further progress with aryl hydrazides in the presence of coupling reagents 1-ethyl-3-(3-dimethyl aminopropyl)carbodiimide hydrochloride (EDCl) and 1-hydroxy benzotriazole (HOBt) in dry THF yielded diacylhydrazines (75). The last step involved thionation of diacylhydrazines with LR, followed by oxidative cyclization in dry THF, leading to good yields of indolyl-1,3,4-thiadiazoles (76). Different solvents, such toluene, dioxane, and xylene, were screened to test the reactions with LR but provided a small amount of product than THF.

The product (76) was purified by column chromatography utilizing ethyl acetate:hexane (7:3) as eluent to afford the pure product (101) [80].

The synthesized novel series of 5-(30-indolyl)-2-substituted-1,3,4-thiadiazoles has displayed cytotoxic activity against various cancer cell lines. Further studies on the SAR are necessary to indicate the role of the heterocyclic linker in indolyl azoles and elucidate their molecular targets.

The most prevalent method for synthesizing 1,3,4-thiadiazoles entails cyclization and dehydration of thiohydrazides or other substrates with the S-C-N-N-C-S moiety. Lawesson’s reagent can perform the thionation of 1,2-diacylhydrazines, including cyclization and dehydrosulfurization, as an improved thiadiazole ring formation method. However, it only deals with a narrow group of compounds, mainly dialkyl thiadiazoles [81]; therefore, Gierczyk and Zalas [82] decided to expand the synthesis of 1,3,4-thiadiazoles, such as mono-, disubstituted, and bis-thiadiazoles and polymers containing the 1,3,4-thiadiazole units.

**Scheme 25:**
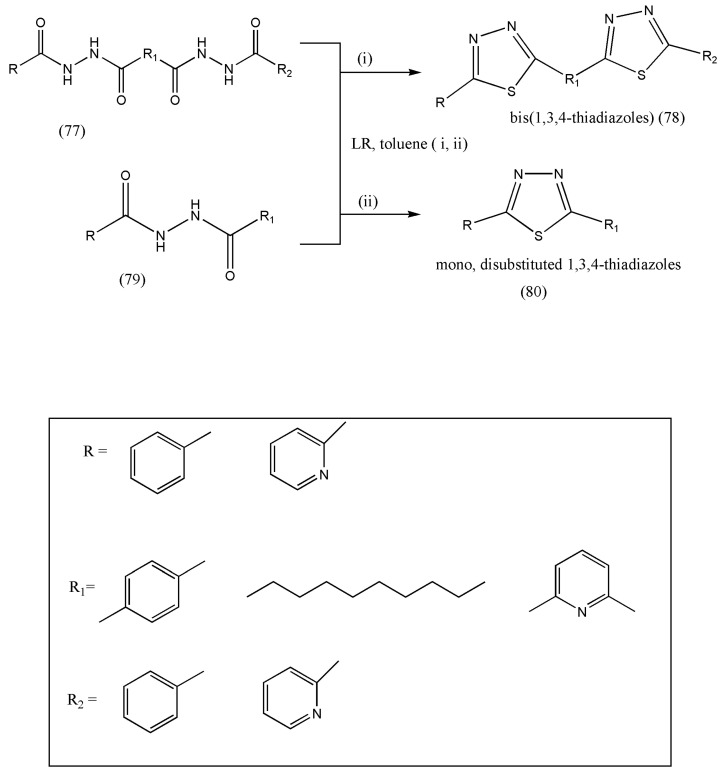
Synthetic pathway to prepare bis-thiadiazoles (78, 80).

Scheme 25 exemplifies the synthesis of different types of substituted 1,3,4-thiadiazoles. Their synthesis with other methods was complex, and the products were acquired in low yield. The methods described by the authors help synthesize mono-, di- (80), and bis-substituted (78) thiadiazoles and polymers containing the 1,3,4-thiadiazole units, which are essential complexing agents and can be used as liquid crystals and conducting polymers. The absence of by-products was observed, making the isolation and purification of 1,3,4-thiadiazoles from the reaction mixture simple.

**Scheme 26:**
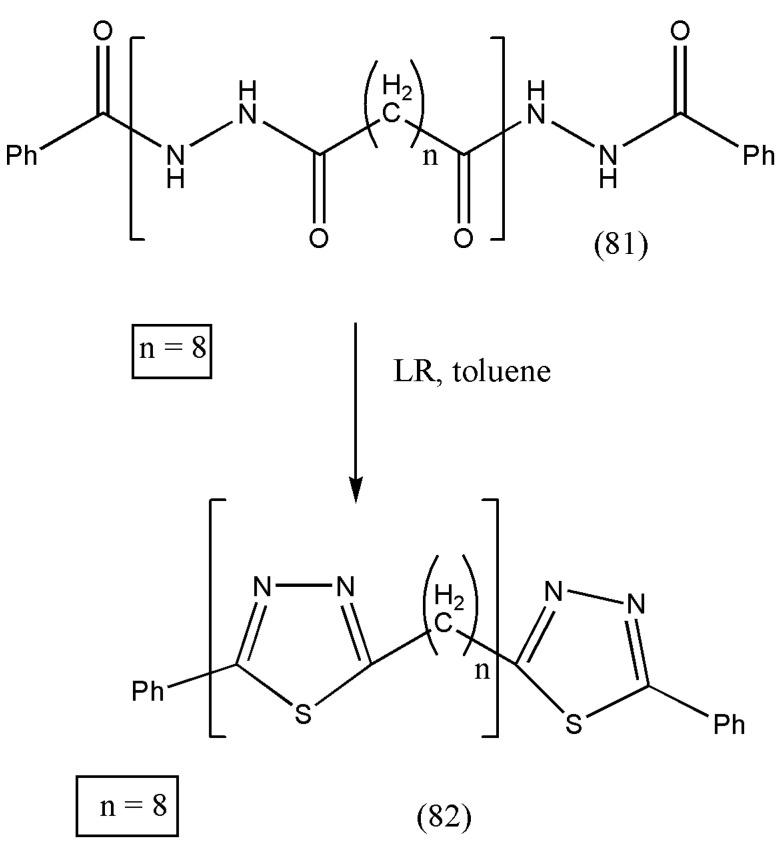
Synthetic pathway to prepare polymer containing 1,3,4-thiadiazole units (82).

The authors went one step further to synthesize polymers containing 1,3,4-thiadiazole units, as explained in Scheme 26. To the suspension of (81) in dry toluene, Lawesson’s reagent was added and heated for 10 h, trailed by evaporating the solvent in vacuo. The crude residue was extracted with diethyl ether, hot dichloromethane, and hot methanol and dried overnight under reduced pressure. The polymer α-(2-phenyl-1,3,4-thiadiazo-5-yl)-ω-phenyl poly(1,3,4-thiadiazol-2,5-diyl-octane-1,8-diyl) (82) was obtained with 90% yield.

The exact mechanism for the formation of synthesized compounds is unknown, but should be studied and applied to synthesize more potent derivatives of 1,3,4-thiadiazoles.

## 8. Synthesis of Biologically Active Thiophenes

The thionation of biomass-derived molecules to S-containing products such as thiophenic acids was first reported by Zheng Li et al. [83]. Thiophene is a five-membered heterocyclic compound, with a significant application in natural products [84,85], pharmaceuticals [86,87], and advanced solar materials [88,89]. The authors created a new class of thiophenes by tandem thionation of levulinic acid (LA) in the presence of Lawesson’s reagent. By tuning the LR:LA mole ratio, an aromatic thiophenic compound, 5-methyl thiophene-2-thiol, was formed as a di-thionated product with many uses as functional materials and in the pharmaceutical industry [83].

The authors articulated that the LR:LA mole ratio plays a vital role in converting levulinic acid to (84), (85), and (86) (Table 3). If the LR:LA mole ratio was 0.5, the main products were (84), (85), and (86), with only a small percentage of (87) at 90 °C (entry 3). With a rise in temperature up to 110 °C, only 2% of LA was converted to (87) at an LR:LA mole ratio of 0.5 (entry 4), whereas a 17% yield of (87) was obtained if the LR:LA mole ratio was doubled at 90 °C (entry 5), which suggests that the LR mainly dominates the degree of thionation of the LA:LA mole ratio.

It was observed that the compound yield (87) increased to 46% at 110 °C in 40 min, and the output further increased to 66% with an increased reaction time of 3 h (entries 6, 7). A marginally lower yield of 63% was obtained when the LA and LR ratios tripled (entry 8). The generation of (87) was substantially enhanced under a higher LR:LA mole ratio of 1.25 as the yield reached 78% within 40 min (entry 9). However, with a further increase of LR:LA to 1.5, no noticeable surge in the output of (87) was witnessed in 40 min (entries 11, 12) [83].

**Table 3:** Optimization of the LR:LA mole ratio at different temperatures (84, 85, 86, 87, 88 structures as in Scheme 27 and C_LA_ = percentage of LA conversion).

Entry	LR:LA	T (°C)	C_LA_%	84%	85%	86%	87%	88%
1.	0.25	90	65	12	21	12	0	0
2.	0.5	70	87	16	18	19	0	0
3.	0.5	90	100	24	52	21	1	0
4.	0.5	110	100	26	55	16	2	0
5.	1	90	100	16	35	24	17	0
6.	1	110	100	6	8	5	46	3
7.	1	110	100	5	4	3	66	6
8.	1	110	100	6	6	2	63	4
9.	1.25	110	100	2	3	0	78	2
10.	1.25	110	100	0	0	0	61	6
11.	1.5	110	100	1	1	0	77	2
12.	1.5	110	100	0	0	0	60	7

The newly derived thiophene (87) is a versatile building block for functional materials, pharmaceutical intermediates, and solvent applications. In addition, the thiophene derivative is also considered a helpful intermediate to prepare various functional groups such as thiophene aldehyde, thiophene carboxylic acid, halothiophene, and thienothiophene.

## 9. Synthesis of Biologically Active Thiazepines

Another class of important heterocyclic compounds for drug discovery is 1,4-thiazepines due to their wide range of therapeutic activities [90]. The benzo-, dibenzo-fused, and oxo derivatives of 1,4-thiazepines are very common in pharmaceuticals and drugs [91,92]. Yilmaz and Zora [93] incorporated conjugated enyne moiety with structural features of 1,4-thiazepines to generate compounds with distinct biological properties. The integration of eyne with 1,4-thiazepines allows their transformation to more complex structures.

In this study, the authors anticipated that thionation of N-(2,4-pentadiynyl) β-enaminones (88) would result in N-(2,4-pentadiynyl) β-enaminothiones (89), which can imitate the chemistry of N-propargylic β-enaminones and produce numerous sulfur-containing heterocyclic molecules.

The thionation of N-(2,4-pentadiynyl) β-enaminones (88) was performed using Lawesson’s reagent. As a result, β-enaminothiones were produced in situ and instantly underwent 7-exo-dig cyclization to afford 2-(prop-2-yn-1-ylidene)-substituted 2,3-dihydro-1,4-thiazepines (90) in one pot (Table 4).

**Scheme 28:**
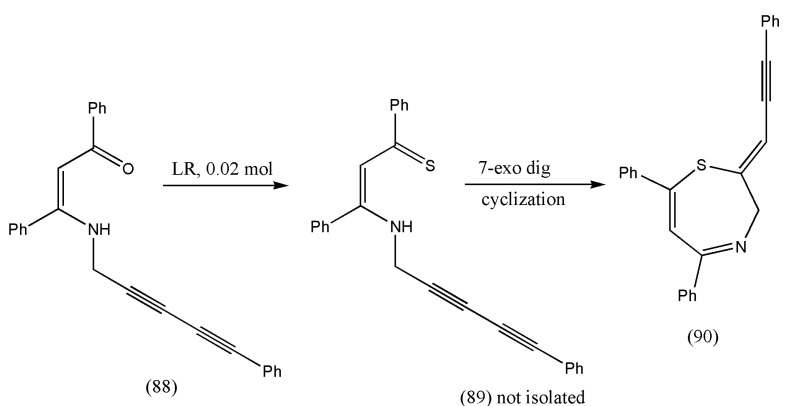
Synthetic pathway to prepare 1,4-thiazepines (90).

Scheme 28 illustrates the synthesis of 2-(2-propyn-1-ylidene)-2,3-dihydro-1,4-thiazepines (90). A solution of N-(2,4-pentadiynyl)-β-enaminone (88) in benzene was added to Lawesson’s reagent. The mixture was heated (65 °C) for 20 min to 3.5 h. The crude product (90) obtained after evaporating the solvent was further subjected to purification via flash chromatography using 9:1 hexane/ethyl acetate as eluent [93].

Due to the existence of conjugated enyne functionality, 1,4-thiazepines can be further expounded into more complex structures. In conclusion, the synthesized 1,4-thiazepines are a pathway for designing new molecular entities and structural leads.

## 10. Synthesis of Biologically Active Phosphine Sulfides

α,β-Unsaturated thioamides have proven to be strong electrophiles and have permitted various challenging asymmetric reactions in the presence of copper(I) catalyst [94].

**Scheme 29:**
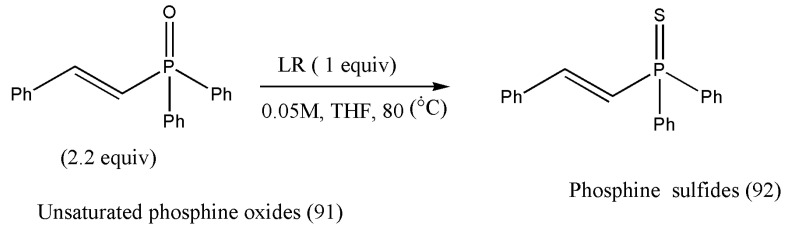
Synthetic pathway to prepare phosphine sulfides (92).

N-Thiophosphinoyl ketimines also allow exceptional copper(I) catalysts to catalyze asymmetric nucleophilic conditions [95]. In 2013, Shibasaki and co-workers [96] reported the synthesis of asymmetric addition of dialkyl phosphite to N-thiophosphinoyl ketimines using copper(I)-catalyst to afford 90% to 96% yields. Recently, in the year 2019, Wang et al. [97] envisioned the preparation of an array of α,β-unsaturated phosphine sulfides from the parent α,β-unsaturated phosphine oxides with Lawesson’s reagent (Scheme 29).

The authors Wang and co-workers initiated the thionation in the presence of LR. Due to the unpleasant odor of LR, phosphine oxide (91) was utilized in 2.2 equiv to attain the complete conversion of LR. The reaction was optimized to get the best yield by using several common solvents such as THF, MeCN, MeOH, DME, toluene, DCM, and DMF (Table 5). THF, MeCN, DME, or toluene reaction produced phosphine sulfides (92) in about 20% yield. With DME as the solvent, the product yield rose (80%) as the reaction temperature was increased to 80 °C. The same trend was detected for the reaction in THF; product (92) was isolated in 96% yield at 80 °C. The product obtained was purified via chromatography using eluents EtOAc/hexane [97].

The designed reaction pathways enjoy a broad substrate scope. Aryls, heteroaryl, and simple and functionalized alkyls can be easily incorporated at the β-positions. Further applications of phosphine sulfides in copper(I)-catalyzed asymmetric reactions should be explored.

## 11. Synthesis of Biologically Active N-Alkylbenzisoselenazolthiones

Ebselen is a synthetic organoselenium drug molecule exhibiting anti-inflammatory, antioxidant, and cytoprotective activity. Recently, it has been projected that ebselen shows inhibitory action against COVID-19 in cell-based assays [98].

Ebselen (N-phenyl-1,2-benzisoselenazol-3(2H)-one) is the first organoselenium compound found to be mimicking glutathione peroxidase (GPx) and with encouraging antioxidant properties. The synthesis of a highly active Se therapeutic that mimics the antioxidant enzyme glutathione peroxidase (GPx) activity remains challenging. Therefore, to improve the antioxidant and anticancer activities of the benzioselenazolone core of the essential ebselen core, several modifications were made by Jacek Ścianowski [99].

The conversion of carbonyl oxygen to sulfur atoms in benzisoselenazol-3-(2H)-one decreases the polarity of the double bond, which significantly influences the stability of the Se-N bond and its reactivity as an antioxidant and the rate of S–Se bond formation (proteins). Based on this hypothesis, a practical synthetic pathway was designed for benzisoselenazol-3-(2H)-thions using LR.

**Scheme 30:**
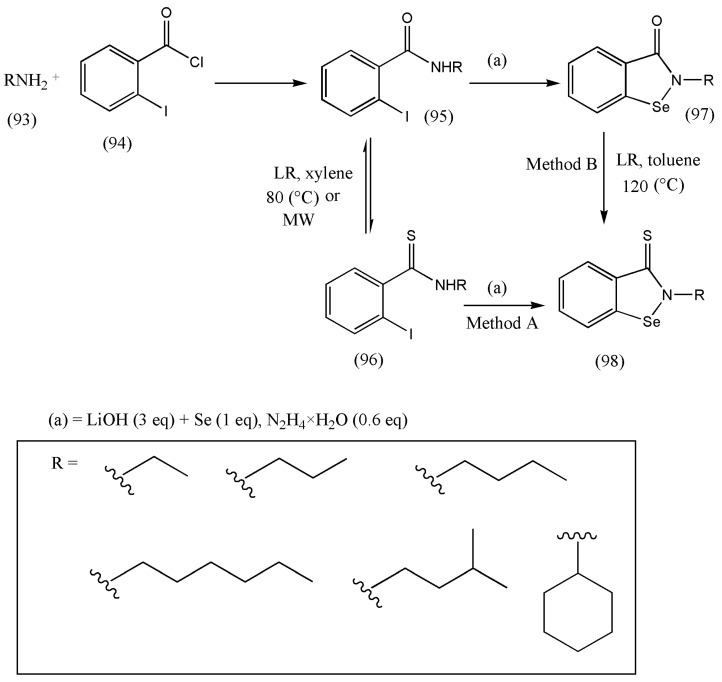
Synthetic pathway to prepare N-alkylbenzisoselenazolthiones (98).

N-alkylbenzisoselenazolthiones (98) were found by two distinct two-step methods (A and B) (Scheme 30). Method A involves N-alkyl-o-iodobenzamides (95) reaction with LR, followed by the nucleophilic substitution of the obtained thioamides (96) with Li_2_Se_2_. Method B was built on the creation of benzisoselenazol-3-(2H)-ones (97) in the reaction of N-alkyl-o-iodobenzamides (94) with Li_2_Se_2_ and then the reaction of ebselen derivatives (97) with LR [99].

Due to the current pandemic situation due to COVID-19, it is necessary to research more drugs as antiviral agents, and since ebselen has inhibitory action against COVID-19, its thio derivatives should also be synthesized and tested.

## 12. Synthesis of Biologically Active Organophosphorus Compounds

Organophosphorus compounds are classified as phosphorus heterocycles, with extensive applications in medicine, agriculture, and industries. Some of their pronounced biological properties include antibiotic [100], antitumor [101], antimicrobial [102], antiviral, and anti-inflammatory [103]. Heterocyclic organophosphorus compounds carrying O–P–S, N–P–S, or O–P–N functionalities have been comprehensively researched for antitumor, anti-inflammatory, and medicinal purposes. The bioactivity of these heterocycles may be attributed to the arrangement of their heteroatom or the size of the ring [104].

**Scheme 31:**
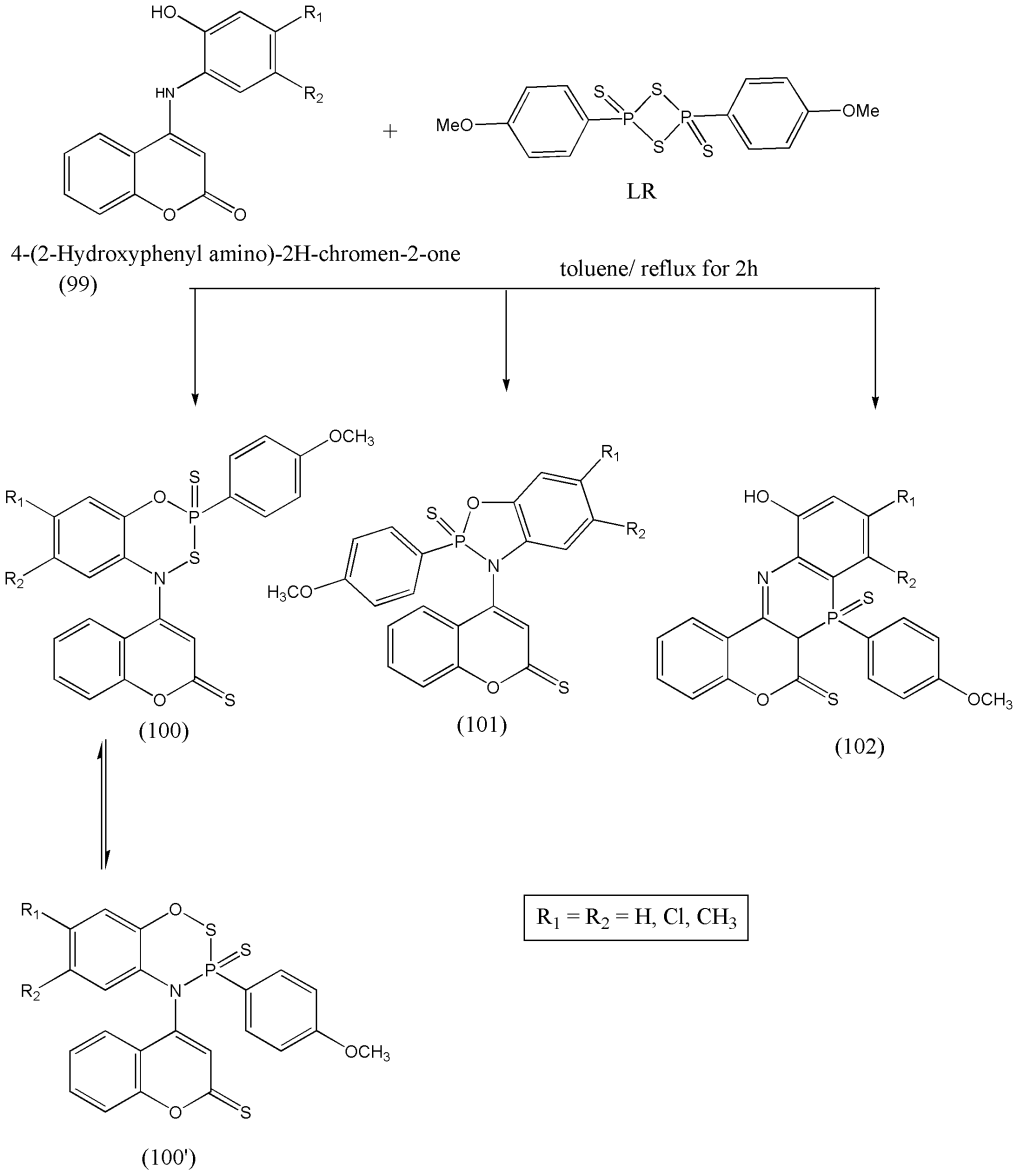
Synthesis of oxathiazaphosphinine (100, 100′), oxazaphosphorine (101), and azaphosphinine (102) merged with thiocoumarin.

Based on the above evaluated biological activities, Zghab et al. [104] synthesized bioactive five- and six-membered phosphorus heterocycles via cyclization reaction of coumarin derivatives with Lawesson’s reagent. The bifunctional coumarin derivatives (99) have several reactive sites that can be utilized as intermediates in synthetic routes for many heterocycles. Therefore, reaction of compound (99) with LR affords five- and six-membered heterocycles with N–P–O, C–N, O–P–S, and S–P–N moiety. The spectral analysis proves the formation of a mixture of three products. The newly synthesized compounds were evaluated for their acetylcholinesterase inhibition. The compounds (100) and (100′) have shown the best anti-AChE activity equivalent to malathion (Scheme 31) [104].

The synthesized compounds have an excellent AChE potency and should be investigated to develop drugs for Alzheimer’s disease.

The previous research of coumarin has explored the low toxicity and remarkable cytotoxic activity of coumarin-based organophosphorus compounds. The effectiveness of coumarin derivatives containing P and S in numerous biological applications prompted Marwa et al. [105] to develop novel diazaphosphinanes coumarin derivatives as diastereomers with cytotoxic and antityrosinase activities.

**Scheme 32:**
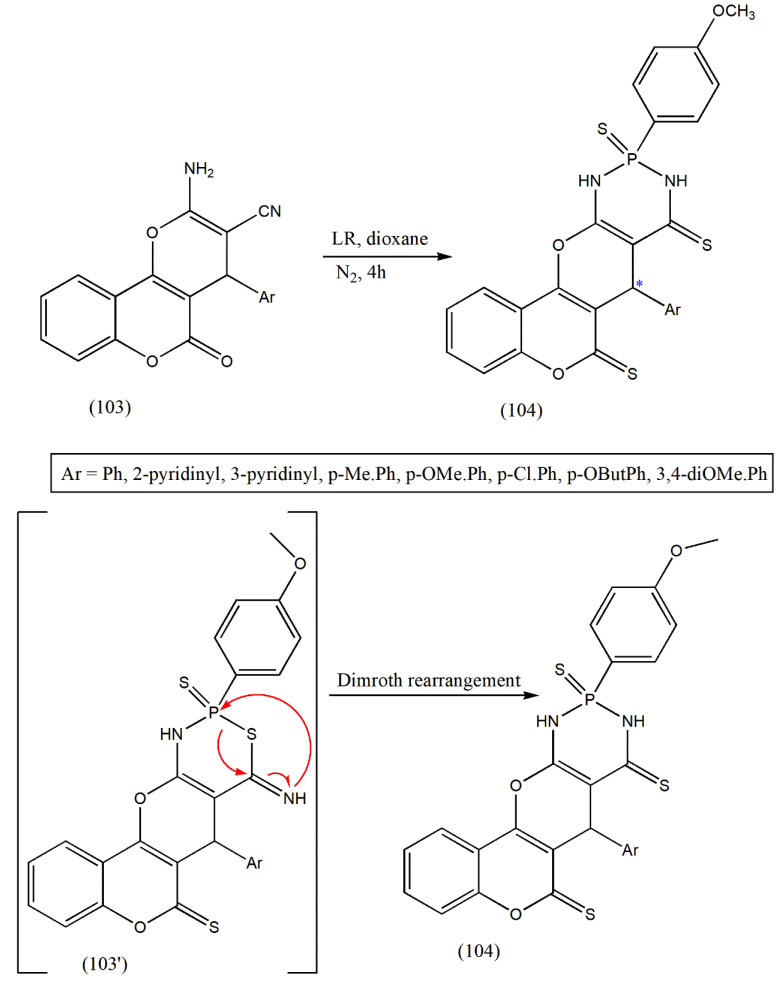
Synthesis of diazaphosphinanes (104).

The coumarin-based α-aminocarbonitriles (103) is a bifunctional precursor treated with LR to produce new coumarinic compounds as diastereomers (104) (Scheme 32). The synthesis of target coumarinic derivatives was achieved in a one-step procedure with LR. The formation of diazaphosphinanes can be explained by the initial thionation of the coumarinic carbonyl, followed by the addition of P-atom of LR and intermolecular cyclization, giving the non-isolable intermediate (103′), which affords fused tetraheterocyclic diazaphosphinanaes (104) via dimroth rearrangement. Most of the synthesized compounds developed a strong capacity of tyrosinase inhibition (% inhibition = 55.02–97.40%), but with moderate cytotoxic activities [105]. The designed compounds should be explored for in vivo activity.

LR can undergo ring closure with substrates containing two functional groups, forming phosphorus heterocycles, with tremendous biological activities such as insecticidal, antibacterial, antifungal, and anticancer pharmacophores. Based on the above findings and further progress in synthesizing bioactive molecules, another class of new fused P-heterocycles, a multi-functional bioactive, was formed via LR’s reaction with pyrano-[2,3-d]pyrimidine-2,4-(1H,3H)-diones by Younes et al. [106].

**Scheme 33:**
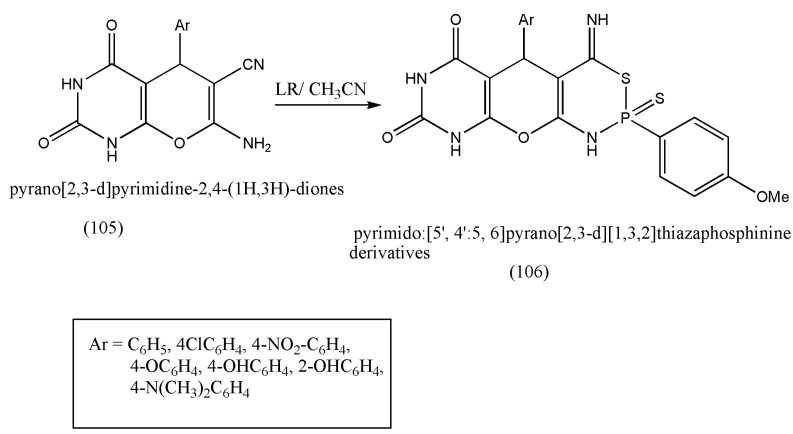
Synthesis of P-heterocycles derivatives (106).

The compound (105) was allowed to react with LR in boiling acetonitrile to produce (106). The reaction was proposed to move via nucleophilic attack of the amino group with LR and SH to the cyano group. All the synthesized compounds displayed antimicrobial activities against bacteria (*Bacillus cereus* and *Staphylococcus albus*) and fungi (dermatophytes fungi) (Scheme 33). This bioactive should be further investigated in pharmaceutical and medicinal fields.

## 13. Synthesis of Biologically Active Steroids and Terpenoids

Steroids and terpenoids are a valuable class of natural organic compounds with fundamental differences in their structural arrangements. Steroids have 17 carbon atoms arranged in four rings and include lipids, naturally occurring sterols, bile acids, sex hormones, some vitamins, and drugs as synthetic steroids, whereas terpenoids belong to a diverse class of synthetic and natural organic compounds derived from hydrocarbon isoprene. Terpenoids include many volatile compounds used in perfume, food flavors, turpentine, carotene pigments, and rubber. The biological significance of steroids and terpenoids is well known and experimented with to replace one or more oxygen atoms with sulfur via a thionation reaction. This replacement is substantial to alter biological properties, which is sometimes quite impactful.

The usefulness of LR as a thionating agent has been reviewed extensively by researchers and, in particular, by Saeed et al. [107] to examine the role of LR to afford thioanalogs of natural steroids and terpenoids.

The addition of heterocyclic rings to steroid molecules can enhance their physiological activity. Thionation is one such possibility that can influence this activity. The introduction of the S atom relative to oxygen increases the reactivity of the thione functional group due to conformational changes in the modified molecule. The previous works on steroid compounds were further modified as biologically active molecules of new thioxosteroid derivatives by Natalija et al. [108].

The authors reported the synthesis of α,β-unsaturated cholestane, androstane, and pregnane carbonyl derivatives (107) with LR under standard conditions. The thionation was also performed under microwave irradiation with a combination of P_4_S_10_/ HMDO in CH_2_Cl_2_.

**Scheme 34:**
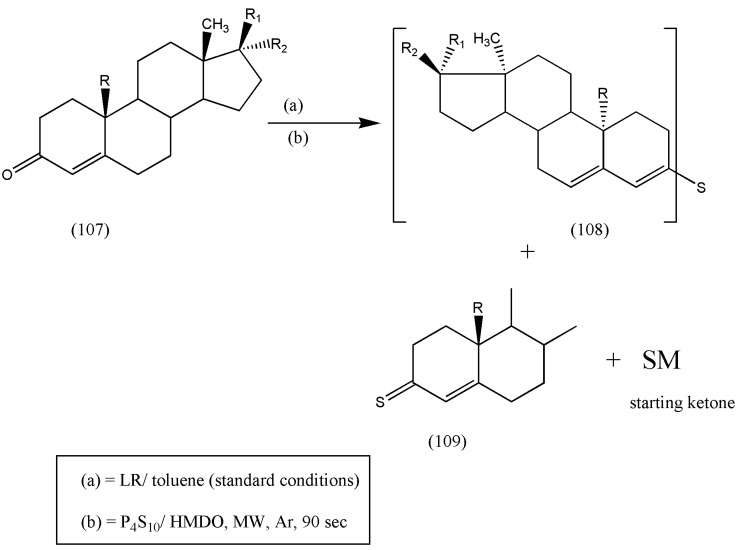
Synthesis of α,β-unsaturated thioketones (109) with LR.

Scheme 34 illustrates the reaction pathway in refluxing toluene for 8 h to form unstable α,β-unsaturated thioketones (109), which dimerizes to subsequent dimer sulfides (108) or decomposes to starting ketones (more stable).

The obtained products were purified via flash chromatography, but partial decomposition to the more stable initial α,β-unsaturated ketones occurred in all cases. Optimization of reaction conditions to eliminate α,β-unsaturated ketones is needed and remains a topic for further investigations. The simplicity of the reaction conditions makes it suitable for chemists in general.

The functions of LR are diverse and can be used to construct five- and six-membered phosphorus heterocycles such as oxathiaphospholes, oxathiaphospholidine-2-thiones, benzothiazoles, and sulfur comprising heterocycles thienothiazines.

**Scheme 35:**
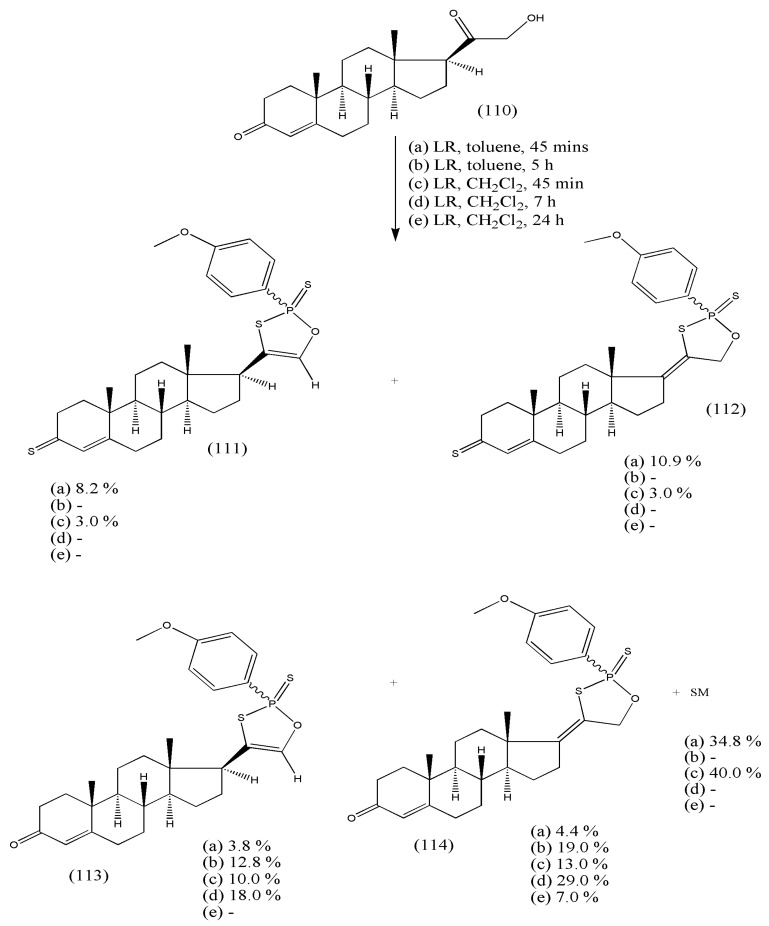
Synthesis of P-heterocyclic androst-4-ene derivatives (111, 112, 113, 114).

The study of steroidal compounds was further extended by Natalija et al. [109] and described the synthesis and configurational analysis of novel steroidal D-ring-substituted heterocycles and evaluated their cytotoxic and antimicrobial resistance properties. Scheme 35 displays the reaction of 21-hydroxyprogesterone (110) with LR in toluene or CH_2_Cl_2_, affording a complex mixture of products, out of which four D-ring-substituted products were, with two tautomeric pairs, isolated and characterized, namely 2-(4-methoxyphenyl)-4-(3-thioxoandrost-4-en-17β-yl)-1,3,2-oxathiaphosphole-2-sulfide (111), 2-(4-methoxyphenyl)-4-(3-thioxoandrost-4-en-17-ylidene)-1,3,2-oxathiaphospholane-2-sulfide (112), 2-(4-methoxyphenyl)-4-(3-oxoandrost-4-en-17β-yl)-1,3,2-oxathiaphosphole-2-sulfide (113), and 2-(4-methoxyphenyl)-4-(3-oxoandrost-4-en-17-ylidene)-1,3,2-oxathiaphospholane-2-sulfide (114).

The two tautomeric pairs are (111, 112) and (113, 114). The synthesized compounds exist as diastereomers in an 8:2 ratio, varying in configuration at the phosphorus atom. All synthesized compounds exercised moderate activity against K562 cells and had antimicrobial activity against Gram-positive and Gram-negative bacteria and fungal cells and toxicity to brine shrimp *Artemia salina* [109]. Thus, additional examinations of structure–activity relationships of these compounds would make it feasible to model novel synthetic antifungal agents.

Although intensive research has been directed towards heterocyclic steroidal compounds, phosphorus heterocycles are still scarce, and only a few steroid derivatives containing phosphorus have been synthesized. The sulfur heterocycles have received widespread attention because of their potent biological activities and pharmaceutical significance [110].

**Scheme 36:**
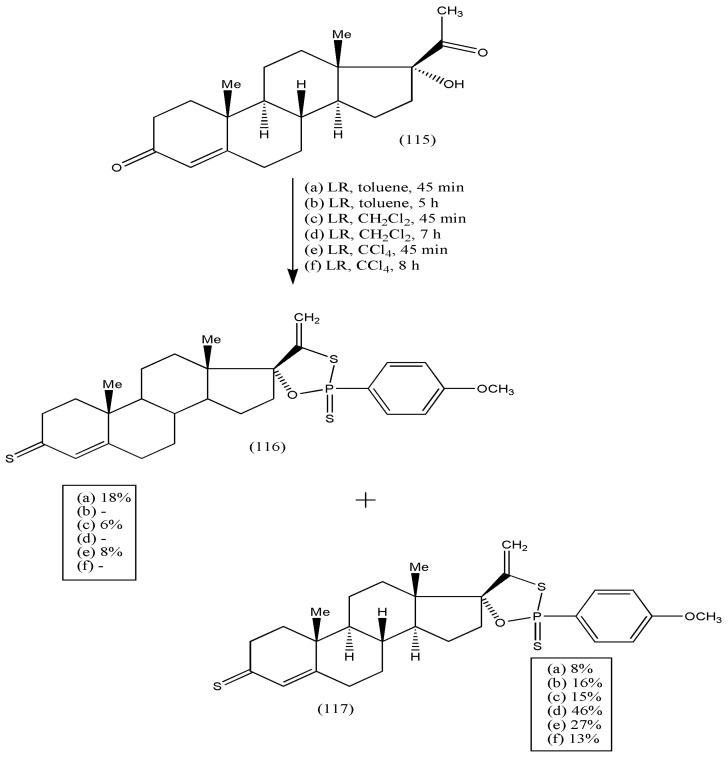
Synthesis of androst-4-en-17-spiro-1,3,2-oxathiaphospholanes (116, 117).

Furthermore, the synthesis of new steroidal compounds was further extended to a new class of androst-4-en-17-spiro-1,3,2-oxathiaphospholanes via the reaction of 17-α-hydroxyprogesterone (115) with LR, used as both thionating and phosphorylating agent [110]. The reactions were performed in different solvents, viz toluene, dichloromethane, and carbon tetrachloride, with varying reaction times. The reaction of (115) with LR afforded a complex mixture of products (Scheme 36), and 3-thioxo-androst-4-en-17-spiro-oxathiaphospholane (116) and 3-oxo-androst-4-en-17-spirooxathiaphospholane (117) were isolated. The yields of the products were optimized by varying solvent/temperature and duration of the reactions. Compound (116) was noticed in each solvent, but quenching of the reaction was required after 45 min and before consumption of (115). Oxathiaphospholane (117) was acquired in all solvents, with the highest yield in dichloromethane for 7 h. Heating for more than 24 h led to the formation of by-products, making the mixture more complex. As a result, isolating desired products was impossible. All synthesized compounds had potent antifungal properties against *Candida albicans* (ATCC 10231) and *Saccharomyces cerevisiae* (ATCC 9763) [110]. The biological activities of the synthesized compounds should be explored substantially via further modifications of these compounds.

Plant triterpenoids are natural compounds and have active antitumor, antiviral, anti-inflammatory, and antiulcer properties among oleanolic, betulinic, and ursolic derivatives and glycyrrhizic acids, among others. The pharmacology and chemistry of glycyrrhetic acid (GLA) have been researched comprehensively. GLA and its derivatives cure allergic skin conditions such as eczema for numerous etiologies, psoriasis, dermatitis, and skin tumor models caused by carcinogens [111,112].

**Scheme 37:**
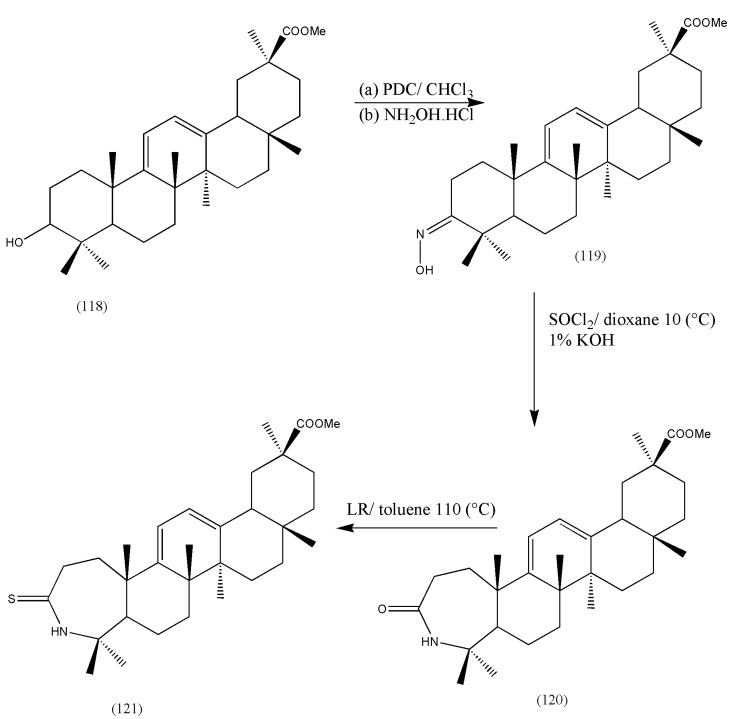
Synthesis of A-homo-4-aza-3-thioxo-18-β-H-olean-9(11),12(13)-dien-30-oic acid (121).

New derivatives of 3β-hydroxy-18β-H-olean-9,12-dien-30-oic acid (118), a GLA analog modified in ring C, were synthesized by Mikhailova et al. [113]. Oxidation of (118) by pyridinium dichromate (PDC) in CH2Cl2, trailed by refluxing in NH_2_OH·HCl in anhydrous pyridine for 1 h, formed 3-hydroxyimine (119). The reaction of (119) with SOCl_2_ in anhydrous dioxane at 10 °C produced (120). The thio analogs (121) were yielded on reaction with LR in toluene for 5 h [113] (Scheme 37). The synthesis of plant terpenoids should be explored for other compounds with potent biological activities. Since plant terpenoids are widely distributed in nature, the ease of availability makes them better candidates for drug discovery.

**Figure 4:**
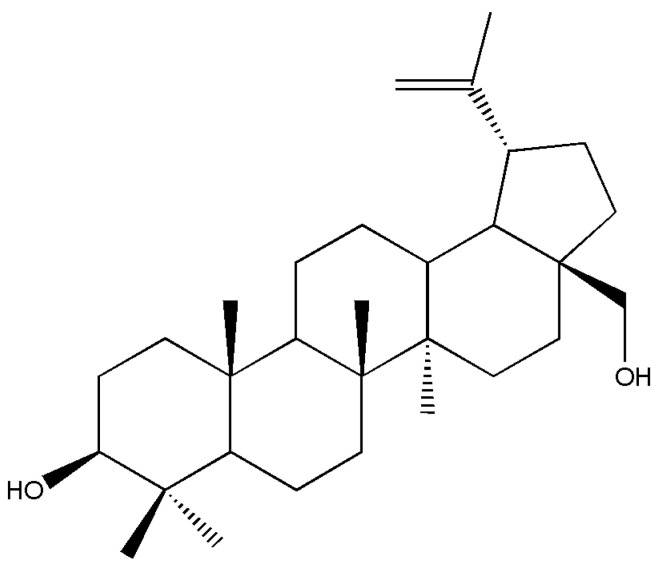
Betulin.

A comprehensive review on pentacyclic triterpenoids with nitrogen and sulfur-containing heterocycles was compiled by Miroslav et al. [114]. The authors synthesized some of the notable reactions of oxotriterpenes with Lawesson’s reagent [115]. Therefore, numerous lupane and 18-α-oleanane carbonyl derivatives were switched to thioanalogs via LR and secondary diketones. The starting triterpenoids were primed from betulin, readily available from birch bark (*Betula pendula*). The betulin (Figure 4) was converted to unsaturated ketone, secondary diketones, hydroxy ketones, ketoacids, anhydrides, and β-ketoester.

**Scheme 38:**
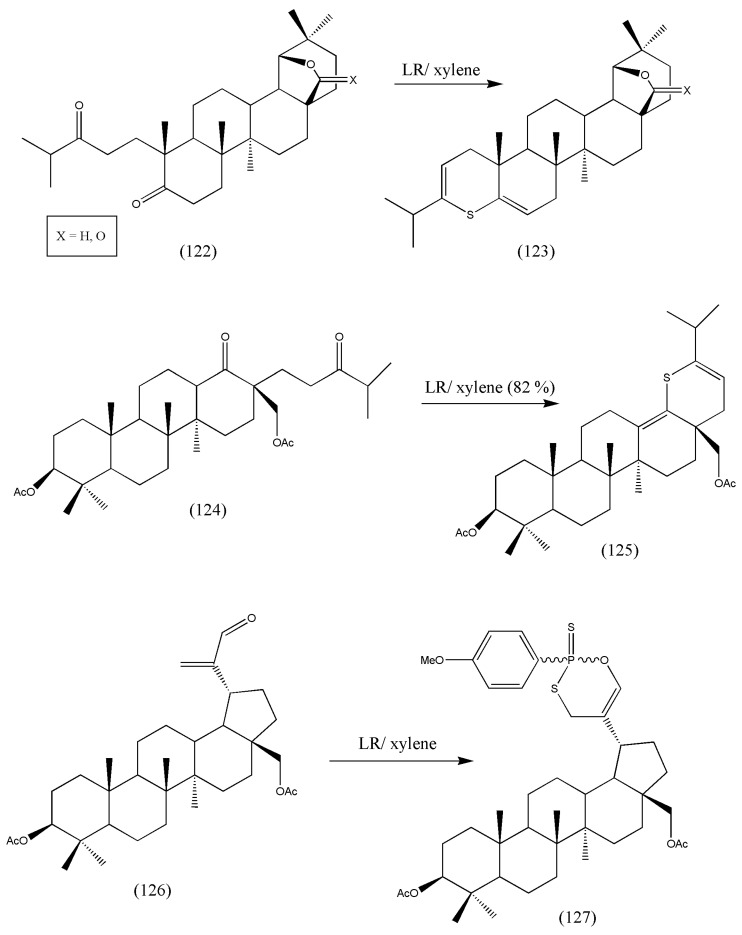
Synthesis of thioanalogs of oxotriterpenes (123, 125, 127).

Further thionation of the derived compounds (122), (124), and (126) was accomplished by LR (Scheme 38). All the synthesized compounds were assessed for in vitro antitumor activity against CEM leukemia cells but failed to have any antitumor activity. Structural modifications of the synthesized molecule may change its biological activity as a potent antitumor. Additional investigations on the structural and biological activities of the synthesized compounds should be conducted.

## 14. Conclusions

Lawesson’s reagent is now a necessary reagent for sulfur chemistry, chiefly for converting almost all types of oxo groups to thio. Like any other reagent, LR is considered a necessary reagent, capable of overcoming barriers and laying colorful feathers to the crown of sulfur chemistry. LR’s fast and slow reactions towards a functional group, such as alcohols, amides, ketones, esters, organophosphorus, steroids, terpenoids, etc., provide synthetic organic chemists with a tool to design their synthetic pathways appropriately. Furthermore, LR is a reagent that can surprise organic chemists with unexpected reactions, leading to new methodologies and reactions. In this review, we emphasized the most recent applications of LR in organic synthesis for thio analogs. With straightforward reactions, we also tried to explain some conversion of thio analogs via biological applications to make the reactions more understandable and leave room for further modifications of their biological properties and organic synthesis with LR.

## Figures and Tables

**Scheme 27:**
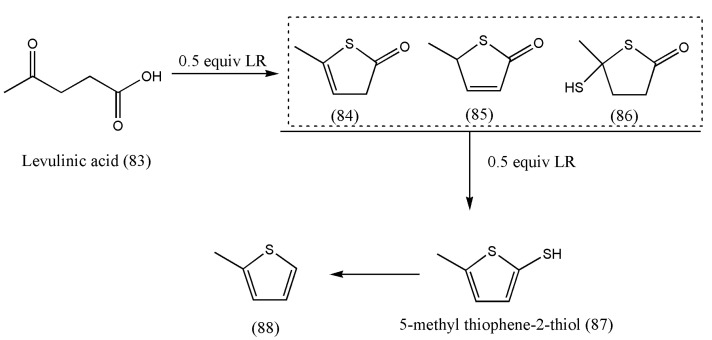
Synthetic pathway to prepare 5-methyl thiophene-2-thiol (87).

**Table 1:** Thionation of quinazolinones bearing various ortho-substituted phenyl groups (R).

Entry	R	A or B	Yields ^a^	ee% ^b^
1.	Me	A	80	83
2.	Et	A	78	95
3.	i-Pr	A	52	97
4.	Cl	A	88	95
5.	I	A	70	99
6.	Me	B	75	93
7.	Cl	B	63	99

Yields ^a^ = isolated yields, and ee% ^b^ was determined by HPLC using a chiral column. A and B are the reaction conditions in the reaction Scheme 6.

**Table 2:** Optimization of alcohol (60) with LR.

Entry	Solvent	T (°C)	Time in Hours	(61) ^a^ %	(62) ^a^ %
1.	DME	25	6	48	29
2.	DME	25	12	31	46
3.	1,4-Dioxane	25	6	18	8
4.	1,4-Dioxane	25	12	27	15
5.	CH_2_Cl_2_	25	6	20	11
6.	CH_2_Cl_2_	25	12	29	20
7.	CH_2_Cl_2_	25	24	39	38
8.	CH_2_Cl_2_	25	48	15	47
9.	CH_3_CN	25	12	25	16
10.	CH_3_CN	80	0.15	43	12
11.	CH_3_CN	80	0.40	61	17
12.	CH_3_CN	80	0.50	32	39
13.	Toluene	90	0.15	75	18
14.	Toluene	110	0.15	56	30
15.	Toluene	110	0.40	38	41

(61 and 62) ^a^ represents isolated yields.

**Table 4:** Optimization of the reaction conditions between N-(2,4-pentadiynyl) β-enaminone (88) and Lawesson’s reagent (LR). Yield ^a^ is an isolated product.

Entry	LR (equiv)	NaH (equiv)	Solvent	Temp (°C)	Time	Yield ^a^ (%)
1.	0.5	-	Benzene	25	20 h	21
2.	0.5	-	Pyridine	25	20 h	No reaction
3.	1.0	-	Benzene	25	20 h	25
4.	1.0	-	Benzene	80	2 h	11
5.	0.5	-	Benzene	65	20 min	58
6.	1.0	-	Benzene	65	20 min	64
7.	1.5	-	Benzene	65	20 min	30
8.	1.0	-	Toluene	65	20 min	25
9.	0.5	0.5	Benzene	25	4 h	6
10.	0.5	1.0	Benzene	65	2 h	36

**Table 5:** Optimization of reaction conditions between unsaturated phosphine oxides (91) and solvents. Yield ^a^ is finalized by ^1^H NMR testing of the crude reaction mixture applying MeNO_2_ as an internal standard.

Entry	Solvents	Temp (°C)	Yield (%) ^a^
1.	THF	RT	26
2.	MeCN	RT	23
3.	MeOH	RT.	-
4.	DME	RT	29
5.	toluene	RT.	19
6.	DCM	RT	8
7.	DMF	RT	-
8.	DME	40	36
9.	DME	60	73
10.	DME	80	82
11.	THF	40	34
12.	THF	60	43
13.	THF	80	96

## Data Availability

Not applicable.
